# Supplementary material for: Democratising deep learning for microscopy with ZeroCostDL4Mic
Source: Nat Commun. 2021 Apr 15;12:2276. doi: 10.1038/s41467-021-22518-0 (PMC8050272; doi:10.1038/s41467-021-22518-0)
Supplement: Supplementary file 1 — Supplementary Information [file 41467_2021_22518_MOESM1_ESM.pdf]

ZeroCostDL4Mic: an open platform to use Deep-Learning in Microscopy

Supplementary Information

Lucas von Chamier[1] *, Romain F. Laine[1,2] *, Johanna Jukkala[3,4], Christoph Spahn[5], Daniel Krentzel[6,7], Elias Nehme[8,9], Martina Lerche[3], Sara Hernández-Pérez[3,10], Pieta K. Mattila[3,10], Eleni Karinou[11], Séamus Holden[11], Ahmet Can Solak[12], Alexander Krull[13-15], Tim-Oliver Buchholz[13,14], Martin L. Jones[6], Loïc A Royer[12], Christophe Leterrier[16], Yoav Shechtman[9], Florian Jug[13,14,17], Mike Heilemann[5], Guillaume Jacquemet[3,4], and Ricardo Henriques[1,2,18]

[1]MRC-Laboratory for Molecular Cell Biology, University College London, London, UK

[2]The Francis Crick Institute, London, UK

[3]Turku Bioscience Centre, University of Turku and Åbo Akademi University, Turku, Finland

[4]Faculty of Science and Engineering, Cell Biology, Åbo Akademi University, Turku, Finland

[5]Institute of Physical and Theoretical Chemistry, Goethe-University Frankfurt, Frankfurt, Germany

[6]Electron Microscopy Science Technology Platform, The Francis Crick Institute, 1 Midland Road, London NW1 1AT, UK

[7]Department of Bioengineering, Imperial College London, South Kensington Campus, London, SW7 2AZ, UK

[8]Department of Electrical Engineering, Technion - Israel Institute of Technology, Haifa, Israel

[9]Department of Biomedical Engineering, Technion - Israel Institute of Technology, Haifa, Israel

[10]Institute of Biomedicine, and MediCity Research Laboratories, University of Turku, Finland

[11]Centre for Bacterial Cell Biology, Biosciences Institute, Faculty of Medical Sciences, Newcastle University, UK

[12]Chan Zuckerberg Biohub, San Francisco, CA, USA

[13]Center for Systems Biology Dresden (CSBD), Dresden, Germany

[14]Max Planck Institute for Molecular Cell Biology and Genetics, Dresden, Germany

[15]Max Planck Institute for Physics of Complex Systems, Dresden, Germany

[16]Aix Marseille Université, CNRS, INP UMR7051, NeuroCyto, Marseille, France

[17]Fondatione Human Technopole, Milano, Italy

[18]Instituto Gulbenkian de Ciência, Oeiras, Portugal

*Equal contributing authors

Supplementary Information: Contents

Supplementary Fig. 1. On the importance of training models on suitable data.

Supplementary Fig. 2. ZeroCostDL4Mic notebooks common workflow.

Supplementary Fig. 3. Graphical user interface (GUI) of the ZeroCostDL4Mic notebooks.

Supplementary Fig. 4. Using alternative cloud computing platforms to run a ZeroCostDL4Mic notebook.

Supplementary Fig. 5. Quality Control in the ZeroCostDL4Mic notebooks.

Supplementary Fig. 6. Model performance vs training parameters.

Supplementary Fig. 7. Example of data augmentation section in the ZeroCostDL4Mic fnet notebook.

Supplementary Note 1: When in doubt, always retrain! A supplementary discussion.

Supplementary Note 2: Quality control of trained models.

Supplementary Note 3: Data augmentation.

Supplementary Note 4: Transfer learning and training from a previously saved model.

Supplementary Note 5: Capabilities of Google Colab.

Supplementary Note 6: Using ZeroCostDL4Mic in alternative cloud-computing platforms.

Supplementary Table 1: Overview of the available datasets used for training the networks.

Supplementary Table 2: Hyperparameters used to train the networks, GPU types allocated and corresponding total training times.

Supplementary Table 3: Install times and training speeds.

Supplementary Table 4: Inference speeds.

Supplementary Table 5: Breaking points for training in Google Colab.

Supplementary Table 6: Breaking points for inference in Google Colab.

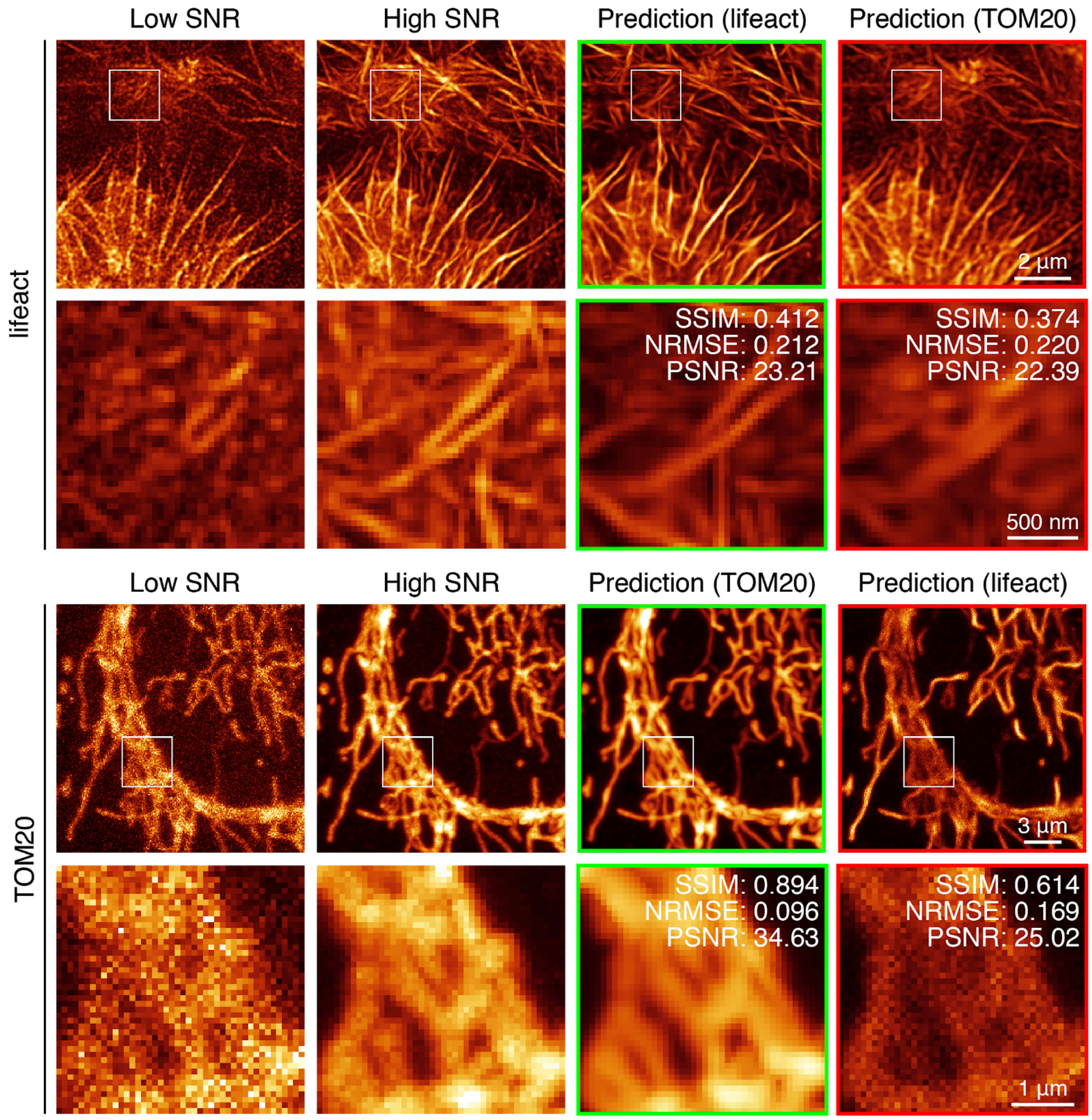

Supplementary Fig. 1. On the importance of training models on suitable data. Top: Filopodia (lifeact) test data and the predictions obtained from a CARE 3D model either trained on similar lifeact dataset (green box) or on TOM20 dataset (red box). **Bottom:** Mitochondria (TOM20) test data and the predictions obtained from a CARE 3D model trained on similar TOM20 dataset (green box) or on the lifeact dataset (red box). Abbreviations used: SNR: signal-to-noise ratio, PSNR: peak-signal-to-noise ratio, SSIM: structural similarity index, NRMSE: normalised root-mean-squared error).

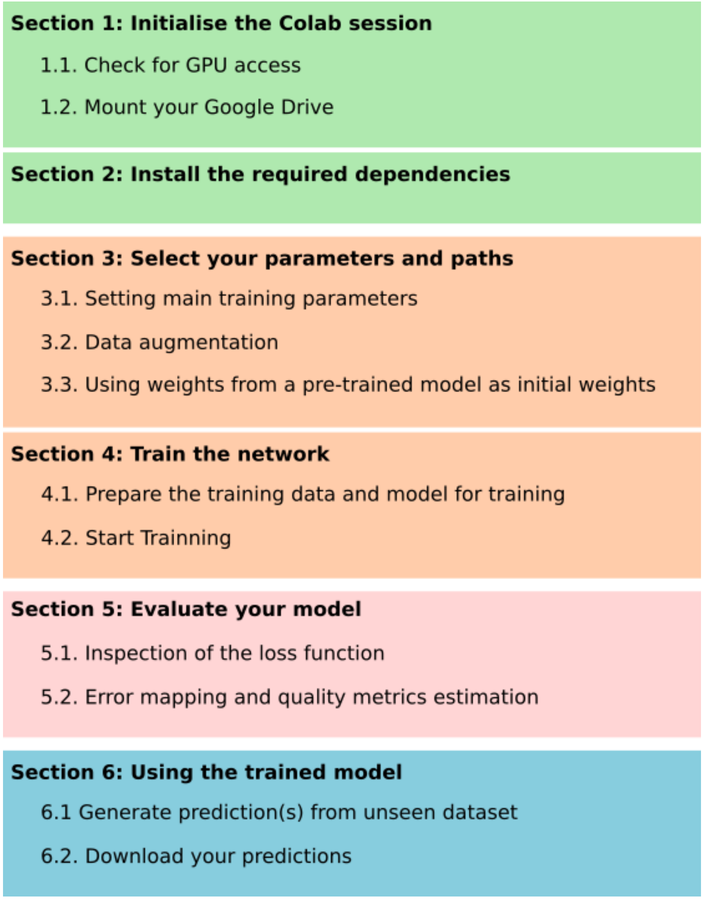

Supplementary Fig. 2. ZeroCostDL4Mic notebooks common workflow.

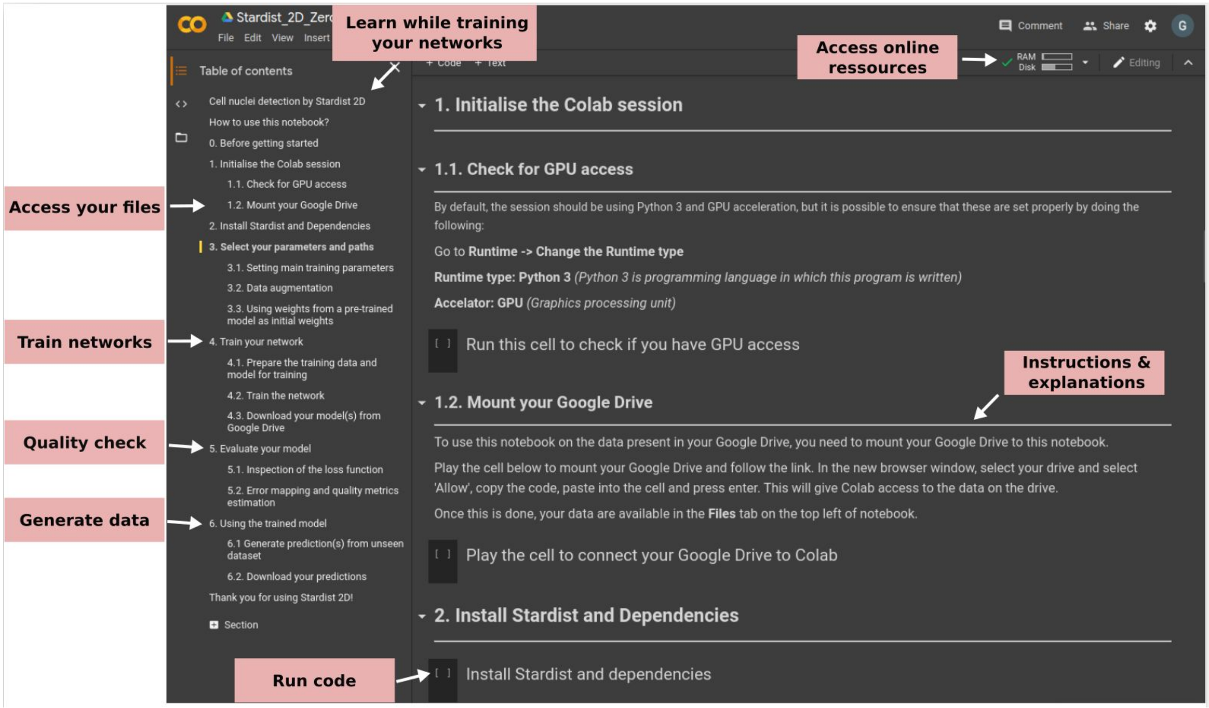

Supplementary Fig. 3. Graphical user interface (GUI) of the ZeroCostDL4Mic notebooks. The layout of the notebook and quick access to the different sections is available on the left panel. The user has access to the files present on Google Drive.

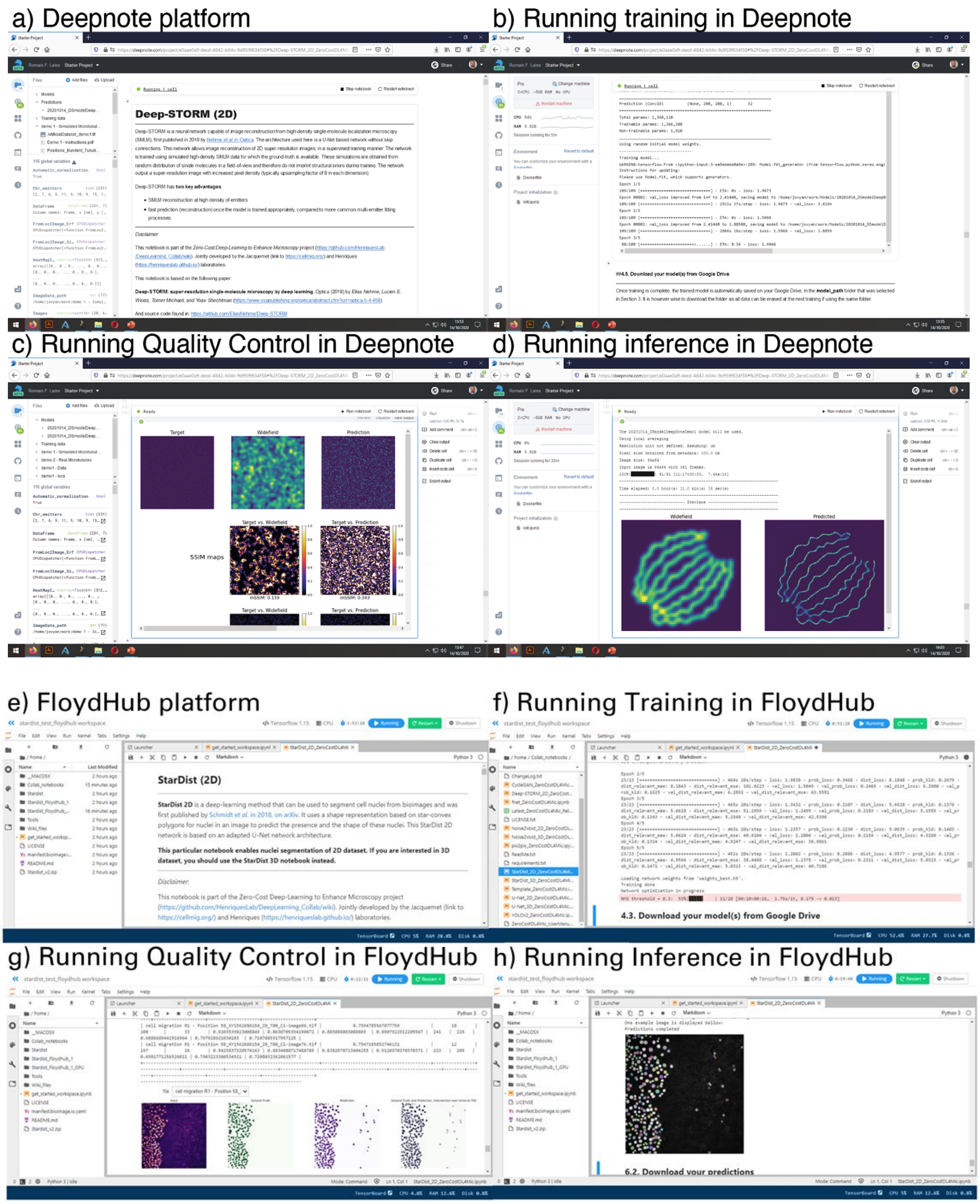

Supplementary Fig. 4. Using alternative cloud computing platforms to run a ZeroCostDL4Mic notebook. Screenshots highlighting the different steps of the DL workflow running within Deepnote (https://deepnote.com) on the Deep-STORM notebook (a-d) and on FloydHub (https://www.floydhub.com/) on the StarDist 2D notebook (e-h).

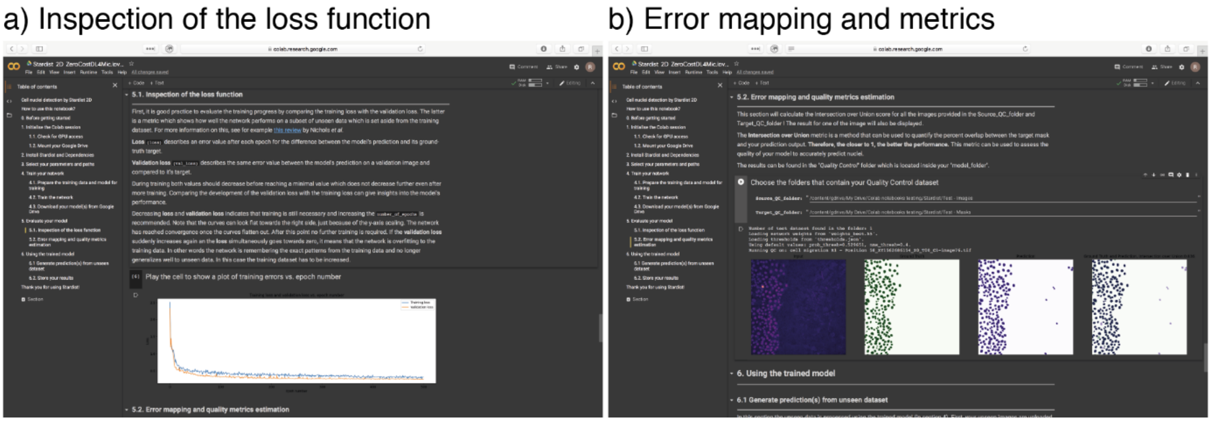

Supplementary Fig. 5. Quality Control in the ZeroCostDL4Mic notebooks. Screenshot of the two quality control steps performed in the StarDist notebook. These quality control sections are available in all of the notebooks that we provide.

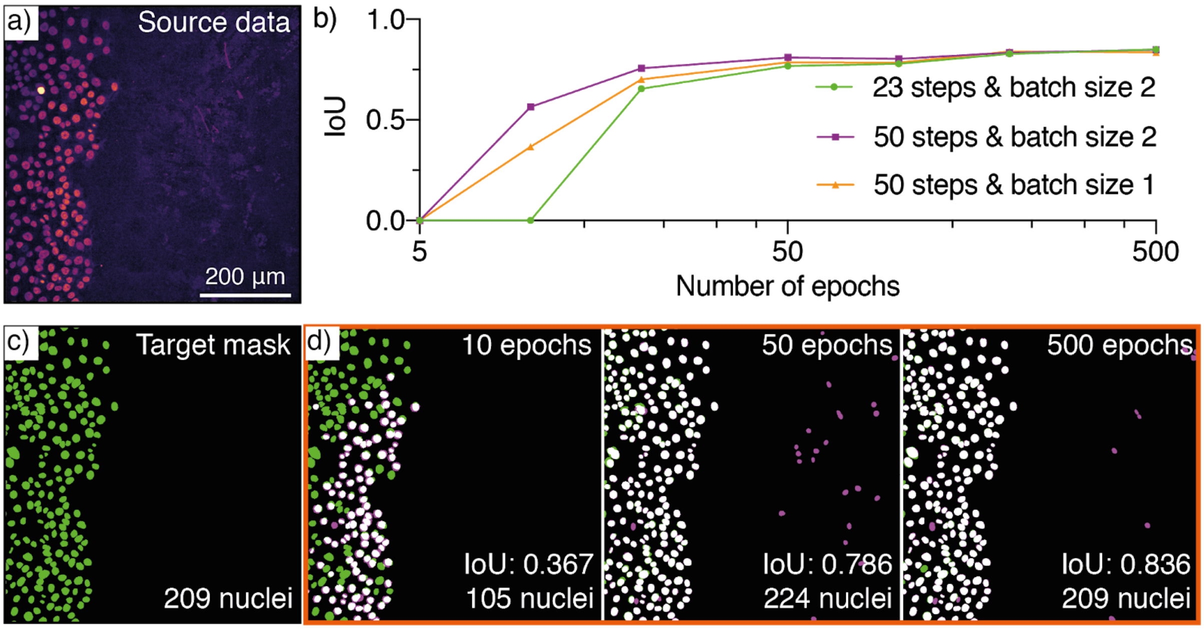

Supplementary Fig. 6. Model performance vs training parameters. (a) Input data: DCIS.COM cells, labelled with SiR-DNA **(b)** The graph depicts an example of how parameter adjustments (batch size, number of steps and number of training epochs) can affect model performance in StarDist nuclear segmentation. **(c)** Hand-labelled ground-truth segmentation mask (Target mask) for input shown in a). The label on the bottom right indicates the number of annotated nuclei. **(d)** Predicted masks for input image a) of three models trained for increasing numbers of epochs with intersection over union (IoU) with respect to c) and detected number of nuclei depicted at the bottom right.

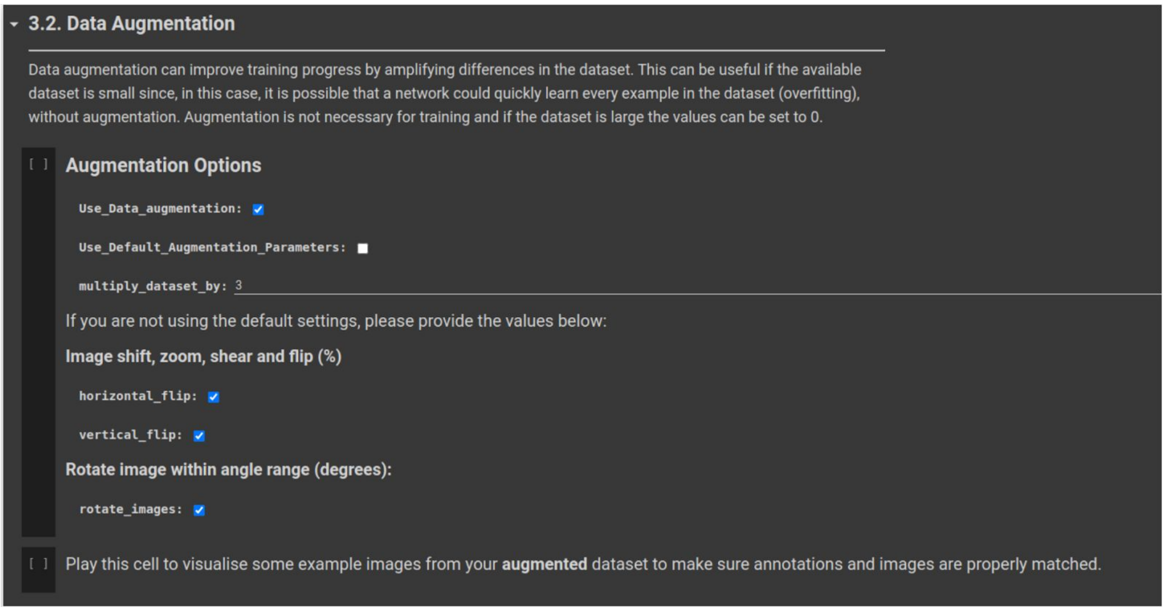

Supplementary Fig. 7. Example of data augmentation section in the ZeroCostDL4Mic fnet notebook. Screenshot highlighting the data augmentation section available in the fnet notebook. Here, only horizontal flip, vertical flip and 90-degree rotations are implemented. Data augmentation can be enabled or disabled in all the provided notebooks.

Supplementary Note 1: When in doubt, always retrain! A supplementary discussion.

The primary focus of ZeroCostDL4Mic is to provide a straightforward and free platform to help novice users in using Deep Learning (DL) in microscopy. A vital component of this platform is the capacity to simplify model training, which remains a significant difficulty. Because it can be challenging to train DL networks (in time, resources, and skills), several labs are taking the approach of providing already trained models, which can then be used to process imaging data[1–4].

Trained models can easily be re-used to analyse data that is very similar to the one used during the initial training. However, pre-trained models should be used with caution on new data as they tend to be very specific to the microscopes and samples used to generate the training dataset. The inappropriate use of pre-trained models can lead to erroneous results when applied to a different dataset type[5,6], which, unfortunately, may lead to visually pleasing yet inaccurate results[7–12]. For example, Supplementary Fig. 1 shows how using an inappropriate model can lead to erroneous results in the prediction. Specifically, a CARE 3D network was trained to denoise either actin (lifeact) or mitochondria (TOM20) data and used to denoise both types of datasets. When using the incorrect model (a model trained on actin to restore mitochondria and vice-versa), the predictions present artefacts and significantly weaker quality control metrics (see Supplementary Note 2 for details on quality control metrics).

Given this issue, it becomes critical for researchers to have the option to train models (or re-train models, using transfer learning, see Supplementary Note 4 dedicated to transfer learning) using their specific data of interest to produce high-fidelity and reliable results.

Supplementary Note 2: Quality control of trained models.

The reliable implementation of DL methods depends on a careful evaluation of the models' output performance; we call this step quality control (QC). QC is crucial to avoid using models that produce low-quality images and artefacts, especially when they may not be easily identifiable by simple visual assessment. These metrics can thus help users improve the models created in the notebooks, for example, by comparing models trained with different hyperparameters or exploring the applicability to data different from the training dataset (generalisation). All the notebooks we provide contain a section dedicated to QC, evaluating the performance of trained models (Supplementary Fig. 5). This section typically has two parts:

- Inspection of the loss function over the number of epochs trained.
- Evaluation of image quality metrics by comparing the model predictions against a ground truth equivalent. Below, we describe these metrics and our implementations in detail.

Of note, the author of CycleGAN and pix2pix does not recommend the visual inspection of the loss function curves to evaluate the training quality achieved with these networks. Therefore, these training curves are not displayed in the pix2pix and CycleGAN notebooks. Instead, these two notebooks save model checkpoints every five epochs; the QC section helps to identify the best checkpoint to use and retrieves the corresponding optimal model. Specifically, these QC sections allow the user to perform predictions using all the saved checkpoints and estimate the quality of these predictions by comparing them to the provided ground truth images using the SSIM metrics.

2.1 Structural Similarity Index (SSIM)

The SSIM metric is used to evaluate whether two images contain the same structures. It is a normalised metric, and an SSIM of 1 indicates a perfect similarity between two images. Therefore, for SSIM, the closer to 1, the better. SSIM is used in the CARE, Noise2Void, pix2pix, CycleGAN and Label-free prediction (fnet) notebooks.

First introduced by Wang *et al.*[13] SSIM is calculated as follows:

$$SSIM(X,Y) = \frac{(2\mu_x\mu_y + C_1)(2\sigma_{xy} + C_2)}{(\mu_x^2 + \mu_y^2 + C_1)(\sigma_x^2 + \sigma_y^2 + C_2)}$$

where X and Y are the images to be compared, μ_x and μ_y the mean pixel values, σ_x^2 and σ_y^2 the variance of pixel values and σ_{xy} the covariance between the pixel values in the images. C_1 and C_2 are constants introduced to avoid instability for small denominators and are defined as:

$$C_1 = (K_1 L)^2$$

$$C_2 = (K_2 L)^2$$

where L is the bit-depth of the images to be compared (for 16-bit images used for the quality assessment in the notebooks $L = 65{,}536$) and $K_1 = 0.01$ and $K_2 = 0.03$, as suggested by the authors of SSIM[13].

To calculate the SSIM on a consistent dynamic range, images are normalised to values between 0 and 1, first by percentile normalisation on all data (source, target, and prediction). Then, both source and prediction are further normalised by linear regression compared to the target.

The percentile normalisation was performed as follows:

$$I_{ij}^{norm} = \frac{I_{ij} - I_{99.9}}{I_{99.9} - I_{0.1}}$$

where I_{ij}^{norm} represents the normalized intensity value at pixel *(i,j)*, I_{ij} the intensity to be rescaled, $I_{99.9}$ the value of the pixel which lies in the 99.9th percentile of pixel values in the image and $I_{0.1}$ the pixel value of the pixel in 0.1th percentile of pixel values in the image. This percentile-based normalisation (instead of using minimal and maximal pixel values) is aimed to prevent the influence of *dead* or *hot* pixels which are common in microscopy images and may distort the useful dynamic range of the image upon normalisation.

The linear regression normalisation is done the same way as in CARE[8], based on least square minimisation, defined as:

$$(\alpha_o, \beta_o) = argmin_{\alpha,\beta} \sum_{i,j} \left(GT_{ij} - \left(\alpha I_{ij} + \beta \right) \right)^2$$

where GT is the ground truth image, *I* the predicted image, GT_{ij} and I_{ij} the respective pixels in the ground truth and prediction and α and β the parameters which rescale the prediction to the dynamic range of the ground-truth.

The normalised image is then calculated as:

$$I^{norm} = \alpha_o I + \beta_o$$

After normalisation, the pixel values above 1 and below 0 are thresholded to 1 and 0, respectively.

Values for SSIM can vary between -1 and 1, with 1 indicating a perfect structural content agreement between the two images. In practice, SSIM values rarely fall below 0, which would represent inverted structures in the image.

For this reason, we truncate SSIM values to a range between 0 and 1. The SSIM map is calculated on a local window around the pixel of interest. The window size is set to 11x11 pixels and a Gaussian weighting function of 1.5 pixels standard deviation. The similarity map obtained displays areas with high and low similarity in different colours, enabling inspection of areas in the image where the model performs better or worse. Therefore, a perfectly-performing model will produce an SSIM map with nearly uniform values close to 1 across the image. A global SSIM metric can also be estimated over the whole image (mSSIM). This is simply determined by calculating the average SSIM value over the whole SSIM map. This value allows an overall quantitative comparison of performance between different images or network parameters. Local SSIM values can be accessed from the SSIM maps that are saved in the QC folder.

In the notebook, the SSIM map is calculated between ground-truth against model prediction and between ground-truth against source image (when appropriate). This way, the metric can be used comparatively to judge the improvement of the prediction over the source image.

The use of SSIM for QC is demonstrated in Fig. 8b, where we compare the prediction of a CARE model trained to denoise fluorescence images of mitochondria (TOM20), with its expected denoised ground-truth. The almost uniform SSIM map and an mSSIM index above 0.9 signify a high level of similarity between the prediction and the ground-truth. It also shows a clear improvement to the SSIM map and index calculated between ground truth and the noisy input image, suggesting that the network improved the input image.

2.2. Root Squared Error (RSE)

RSE is used in the CARE, Noise2Void, pix2pix, CycleGAN and Label-free prediction (fnet) notebooks. The RSE is obtained by calculating the absolute difference between two images on a pixel-per-pixel basis. By calculating the RSE between the prediction and the target image (ground-truth), this error map shows areas of high and low errors in different colours, providing a second metric for inspecting local artefacts.

$$RSE_{ij} = \sqrt{\left(GT_{ij} - I_{ij}^{norm}\right)^2}$$

Similarly to SSIM, we also provide an image-wise metric as a normalised root mean squared error (NRMSE) between the images defined as:

$$NRMSE = \sqrt{\sum_{i,j}\left(GT_{ij} - I_{ij}^{norm}\right)^2}$$

The RSE map and NRMSE reflect the amount of discrepancy between two images. In this case, good performance is indicated by low values for these metrics. A perfect agreement with the ground truth image will lead to RSE = 0 across the image and NMRSE = 0.

In the notebooks using RSE, a comparison between prediction against ground-truth and source against ground-truth is shown side-by-side to indicate how much the image has improved (i.e. brought closer to the ground-truth than the source) by the trained model. The output of the RSE metric is also shown in Fig. 8b, where we compare the prediction of the trained CARE network with the expected ground-truth. The RSE map is almost uniformly dark, and the NRMSE score is low, suggesting a good match between ground truth and prediction. The comparison with the same metrics calculated between the noisy input and the ground truth also shows how the CARE prediction improved the image by reducing the errors seen in the RSE map and by lowering the value of the NRMSE compared to the input image.

2.3. Peak signal-to-noise ratio (PSNR)

PSNR is a metric typically used to quantify the performance of image compression algorithms. It is based on the RSE metric and provides a measure for noise corruption based on the decibel scale. High PSNR values indicate a low contribution of noise, while low PSNR values indicate the opposite. It is hence well suited to interpret the performance of denoising DL approaches. In ZeroCostDL4Mic, we used PSNR for denoising and image-to-image translation tasks, where GT data is available (CARE (Fig. 4), fnet, cycleGAN and pix2pix (Fig. 6). The PSNR ratio is calculated as follows:

$$PSNR = 20\ log_{10}(L) - 10\ log_{10}(MSE)$$

where L represents the maximum pixel intensity of the images to be compared, generally defined as $L = 2^B-1$, where B is the bit-depth of the images, and MSE represents the mean squared error between the GT and predicted image, defined as:

$$MSE = \frac{1}{n}\sum_{i,j}\left(GT_{ij} - I_{ij}\right)^2$$

Where n is the number of pixels in the image and $i, j,$ GT and I are defined as above.

2.4. Intersection over union (IoU)

For the U-Net and StarDist segmentation networks, we use the intersection over union (IoU) metric, commonly used for segmentation performance evaluation[14].

To test how well the model segments an input image the ground-truth mask is compared to the predicted segmentation mask by dividing the number of pixels shared between both masks by the total number of pixels in the union of the two masks:

$$IoU = \frac{I \cap GT}{I \cup GT}$$

where I represents the predicted image and GT the ground-truth image.

The IoU metric quantifies the percentage of overlap between the target mask and the prediction output. Therefore, scores closer to 1 typically mean better performance of the trained model.

How this metric is used for quality control is shown in Fig. 8c. The overlay of ground truth (target) and prediction can act as a direct visual readout of the IoU score. Here, the white areas represent where the signal predicted by the StarDist network overlaps with the target, therefore agreeing with the ground-truth. A large proportion of white areas in the overlay suggests a good agreement between ground truth and prediction, which is also reflected by a high IoU score of over 0.8.

While using these metrics as QC for the models cannot prevent errors or artefacts from occurring in the prediction, they can characterise the model's performance. This can be exploited to improve model performance, as shown in Supplementary Fig. 6. The figure shows an example of how the IoU score in StarDist can aid in identifying a set of training parameters that can accelerate how quickly the model reaches its top performance. Here, we show how the number of epochs, number of steps, and batch size can affect the IoU score. Additionally, the number of nuclei found in the image also provides a way to assess the model performance compared to the number of nuclei identified from the ground-truth (target) mask.

2.5. Mean average precision (mAP) score

In order to quantify the performance of the YOLOv2 models obtained with ZeroCostDL4Mic, we estimate the average precision (AP) of the model on test datasets, as is commonly done to evaluate object detection networks[15,16]. The AP metric encapsulates both how well the model identifies objects in an image and how accurate its class predictions are.

When making predictions, an object detection model will give three outputs per object: the object's bounding box coordinates (identifying the location of the object within the image), its predicted class (what type of object it is) and the confidence (the probability of the class being accurate as estimated by the model for that particular object). The first step to calculate the AP is to rank all object detections of a class by confidence (highest confidence first).

The resulting list is then used to calculate the precision and recall parameters with an increasing number of detections taken into account in the ranked order. The precision and recall metrics are defined as follows:

$$Precision = \frac{True\ Positives}{True\ Positives + False\ Positives}$$

$$Recall = \frac{True\ Positives}{True\ Positives + False\ Negatives}$$

In our case, a true positive detection is defined as a detection with the correct class and where the bounding boxes predicted by the model overlap sufficiently with that of the ground-truth annotation for that object. We defined a sufficient level of overlap when the IoU metric (defined in Section 2.3) between ground-truth and predicted bounding boxes reaches a minimum threshold value. By default, in our notebook, this threshold value is set to 0.3.

For each class, the precision and recall scores are then evaluated by including an increasing number of detections following the ranked order, i.e. from highest to lowest confidence. Thus, the precision of a trained model usually begins with values near 1 and tends to drop as more false positives are encountered in the list of detections (lower confidence). In contrast, the recall value measures the proportion of true positives in all possible positives in the dataset which means that it will tend to increase as the number of true positives increases since more detections are taken into account with each object on the list. When these values of precision and recall estimated in ranking order are plotted against each other, this results in a characteristic p-r (precision-recall) curve[17] (see Fig. 8d for examples of p-r curves).

This plot aims to represent the typical trade-off between precision and recall (or rather between false positive rate and false-negative rate), since typically, precision will drop as recall improves. One way to interpret the curve is the following: the higher the p-r curve as the recall increases, the better the performance of the model. A perfect model will display precision of 1 all the way to a recall value of 1. **Therefore, the area under the curve represents a good way to estimate the overall precision of the model for that particular class**. The AP metric essentially describes the area under the curve of the p-r plot. In our notebook, the AP is calculated as outlined in the PASCAL VOC protocol[18] using a

simple interpolation in the precision scores, such that the precision at recall r is equal to the maximum precision for any $r' \geq r$. This reduces the effect of individual detections in the data on the AP[15].

Since recall and precision are values with a range of 0 to 1, the AP value also varies from 0 to 1 where values closer to 1 indicate a better performance of the model on a given object class.

The mean average precision (mAP) of a YOLOv2 model takes into account the AP of all object classes as follows:

$$mAP = \frac{1}{n}\sum_{i}^{n} AP_i$$

where n is the number of classes in the dataset and AP_i is the average precision of a specific class i.

The p-r curve and the AP score for each class as well as the mAP value are all calculated and presented to the user as output in the YOLOv2 notebook QC section. Examples of p-r curves are shown in Fig. 8d for the most common cell shape, *elongated*, as also shown in our example dataset (see also in Fig. 3). The p-r curves offer a performance comparison between models trained without and with 8x data augmentation (see Supplementary Note 3 for details about data augmentation). The performance of the model significantly improves with 8x data augmentation, as highlighted in the p-r curve by the higher level of precision across the range of recall and the corresponding AP values compared to the non-augmented dataset (Fig. 8d).

For an additional visualisation of the QC, the bounding box coordinates for both ground-truth and predicted labels are also saved in .csv files and can be plotted after the quality control step, e.g. using ImageJ.

2.5. F1-score

Although the AP score is widely used as a metric in object detection research and challenges[19] there are well-known drawbacks to this metric too[1]. In the context of the object detection performed in the ZeroCostDL4Mic notebooks one disadvantage of using AP alone to measure the model predictions' quality is that it can disguise poor model performance at the threshold usually required for applications of the model. For instance, models with a high rate of true positive detections but poor overall sensitivity, i.e. high number of false negatives (initially high precision with a steep drop on p-r curve) could have the same AP score as a model which may have more false positives but much fewer false negatives (lower precision with a gradual decline on p-r curve). The difference can be significant in practice, e.g. if the model is trained to identify a pathogen or cancer, where false negatives could be

more detrimental than false positives. Hence, the AP alone can give an incomplete picture of the model's performance.

We, therefore, additionally calculate the F1 metric, which gives an indication of how balanced the precision and recall of a model's predictions are[21]. The F1 score is calculated as the harmonic mean of precision and recall:

$$F1 = 2 \frac{Precision\ x\ Recall}{(Precision + Recall)}$$

where precision and recall are defined as above. The F1 metric gives equal weight to the precision and recall values. As the highest values for precision and recall are 1 the best possible score for F1, i.e. when the false positive and false negative rates over the entire test dataset are zero, is also 1.

In the YOLOv2 notebook, the F1 score is shown to the user together with the AP score at the end of the QC section. For the user, it is useful to see both scores since the F1 metric takes into account only the total values of precision and recall (after all predictions are evaluated) whereas the p-r curves which are used to calculate the AP score can give an indication of the model's performance across different levels of recall.

2.6. Other metrics used in the StarDist notebook

In the StarDist notebook, the IoU is both calculated over the whole image and on a per-object basis. The value displayed in the notebook is the IoU value calculated over the entire image (as defined in 2.3). This score is then used to calculate the other metrics available in the StarDist notebook.

"n_true" refers to the number of objects present in the ground truth image. "n_pred" refers to the number of objects present in the predicted image.

When a segmented object has an IoU value above 0.5 (compared to the corresponding ground truth), it is then considered a true positive. The number of "true positives" is calculated in the StarDist notebook. The number of "false positive" is then defined as "false positive" = "n_pred" - "true positive". The number of "false negative" is defined as "false negative" = "n_true" - "true positive".

Additionally, as has been recently suggested in studies on nuclear segmentation[22,23] we calculate the F1 score which is displayed together with precision and recall of the predictions vs the ground-truth annotations (F1, precision and recall calculated as in 2.4 and 2.5).

The "mean_matched_score" is the mean IoUs of matched true positives. The "mean_true_score" is the mean IoUs of matched true positives but normalised by the total number of GT objects. The "panoptic_quality" is calculated as described by Kirillov *et al.*[24].

Supplementary Note 3: Data augmentation.

Data augmentation is a strategy used to artificially increase the size of training datasets. It commonly consists of applying a set of spatial transformations to both source and target data in the training dataset, such as rotation or vertical/horizontal flipping. Still, more complex transformations can also be used, such as shearing or zooming. Simply flipping (horizontal and vertical) and rotating all the images in a dataset by 90 degrees will increase the size of a dataset by 8. Data augmentation may improve the generalisation of a model by amplifying diversity in the dataset. This may be especially useful if the available dataset is small, which can occur if it is expensive or time-consuming to generate. All ZeroCostDL4Mic notebooks contain the possibility to enable or disable data augmentation (Supplementary Fig. 7), but its implementation strategy is adapted to each notebook. For instance, we took advantage of the Augmentor library[25] in the StarDist 2D, pix2pix and CARE 2D notebook, while simpler augmentation strategies such as flipping and rotation are implemented in the CARE 3D and fnet notebooks. In the 3D U-Net and the 3D StarDist notebooks, we also implemented the "elastic deform library" (https://pypi.org/project/elasticdeform/). A different augmentation library (imgaug) is used to augment images and bounding boxes in the YOLOv2 notebook (https://github.com/aleju/imgaug)[26].

Importantly, data augmentation can also be detrimental to the training process and lead to the generation of artefacts. So, we recommend that networks be trained with and without augmentation, and for the user to use the QC section available in our notebooks to assess if using data augmentation leads to performance improvements. For instance, when training YOLOv2, with our test dataset, we found that the model performance considerably improved when performing data augmentation on the training images and bounding boxes by flipping and successive rotations by 90 degrees, as quantified by the mAP, shown in Fig. 9 and the respective p-r curves shown in Fig. 8d. In practice, YOLOv2 models trained on datasets with increasing augmentation factors become more sensitive to the cells in the image and improve bounding box positioning around detected objects.

Supplementary Note 4: Transfer learning and training from a previously saved model.

The performance and generalisation of DL networks often scale with the size and diversity of the training dataset. Because of this, trained models often reach peak performance when generated using large training datasets. Importantly, training models with large training datasets require vast amounts of computational power, making them expensive to produce (which in some cases may not be possible within ZeroCostDL4Mic, see Supplementary Note 5 for details on the resource available with Google Colab). Because of this, training very general models is currently limited to computer science developers with access to large resources. On the other hand, individual scientists may want, instead, to train DL models that are high-performance but specific for their data.

However, when publicly available, DL models trained on large amounts of data can be extremely valuable. On the one hand, such models can be directly used by users to perform predictions. This can have several downsides, and it is not something we would generally recommend (see Supplementary Note 1 for discussion on the topic). On the other hand, a very efficient way to take advantage of these while retaining good performance for the specific data of interest is to use these trained models as a starting point for training a new DL model.

Indeed, an already trained model, when trained on large amounts of related data, will contain useful features that can be reused to speed up the training of another model. Therefore, it can be beneficial to use these trained models as a starting point for training a DL network instead of starting from a blank model (training from scratch), where all the model weights are typically initialised to randomly allocated values. So, initialising the model weights to those of a trained model is a powerful approach known as transfer learning[27] and is now common practice in the DL field. Transfer learning can improve performance (necessitating fewer epochs) and generate higher-quality models than those trained from scratch[28].

As we believe that transfer learning is a compelling strategy to generate high-quality DL models, we implemented the possibility to perform transfer learning in all of our ZeroCostDL4Mic notebooks. Indeed, users can quickly load trained model weights and re-training these models on custom data (Fig. 9d-e). Already trained models of broad interest can even be directly downloaded within our notebooks.

To illustrate the performance improvement that can be achieved using transfer learning, we compared the results obtained by training a StarDist model from scratch to the result obtained when an already trained model is used as a starting point. The trained model we choose is the *2D_versatile_fluo* StarDist model made available by the StarDist authors[2,14].

This model was generated using data related (images of nuclei) but distinct from our own and is starting to be widely used by the community, to perform prediction, via its integration to the StarDist Fiji plugin[8,9]. Importantly, using the *2D_versatile_fluo* model to perform prediction on our data led to nuclei segmentation but also to the generation of large artefacts rendering it unusable on its own (Fig. 9d-e). Typically, to obtain high-quality predictions from models trained from scratch, we needed to train our StarDist models for more than 200 epochs (Fig. 9d-e). However, when using the *2D_versatile_fluo model* as a starting point via transfer learning, very high-quality prediction can be made using a model trained for as little as 5 epochs (Fig. 9d-e).

Transfer learning also allows the user to circumvent the Google Colab 12h training time limit (see Supplementary Note 5 for discussion on resources available with Google Colab). Loading a pre-trained model for training also enables training to occur over multiple Colab runtimes, as well as to do transfer learning using models trained outside of ZeroCostDL4Mic. We believe that transfer learning is an especially attractive feature of ZeroCostDL4Mic as it allows users to tune generalist models to their data and generate optimal results while requiring minimal resources and time. Therefore we encourage developers and users to share their trained models via the emerging online "model zoo" so that they can be used by others to enable faster re-training.

Supplementary Note 5: Capabilities and limitations of Google Colab.

The Google Colab (https://colab.research.google.com) platform offers free and easy access to a Graphical Processing Unit (GPU) and Tensor Processing Unit (TPU), which enables users to train many networks on bespoke training datasets, as well as running predictions on unseen data. In practice, for each notebook session, Google Colab assembles a virtual machine with allocated RAM, disk space, and access to GPU/TPU. Although these resources are free, they are finite. It is important to consider the available resources when exploiting Google Colab for DL training and predictions. In our experience, we consistently found that GPU acceleration provided faster computation than TPU for the networks and datasets presented here. Therefore, we focused our attention on GPU accessibility and performance below. In the following sections, we discuss how to handle these resources in order to perform efficient training.

5.1. Google Drive storage

When using a free Google Drive (https://www.google.com/drive/) account to perform training, the user will have access to 15 GB of data storage that can be accessed by any Google Colab notebook. All training and test datasets, plus the output of the training, need to fit within this 15 GB limit. We have shown, however, that this is sufficient to efficiently train all of the networks with the datasets that we provide (see Supplementary Table 1 for details about the datasets). Additional storage space can also be purchased from Google.

5.2. Remote RAM capacity

A 12.72 GB RAM limit currently exists for the free GPU or TPU provided by Google Colab. The amount of RAM required to execute the code is determined by the size of the training data, the number and sizes of patches/batches. Exceeding this RAM limit can cause the notebook to crash when initialising the networks. For the datasets we have tested, it was always possible to train the networks with the currently available RAM. It is important to note that large datasets (e.g. datasets made of large 3D stacks) may reach or exceed the RAM capacity when using a large number of patches/batches or data augmentation.

5.3. Time-outs

12-hour time-out. The time taken to train a network sufficiently is primarily determined by the size of the training dataset, the number of steps/epochs/patches, and the efficiency of the underlying code. Google Colab currently offers 12 hours of GPU/TPU access after which remote data loaded into the virtual machine will be lost, a limit primarily enforced to prevent cryptocurrency-mining. For users, this means that if training has not completed by the 12h limit, it will be stopped. This constraint can be annoying when networks need to be trained over many epochs to reach high performance, often necessary for large datasets. However, users can easily circumvent this issue by continuing their training sessions over multiple Colab runtime. Indeed, in all ZeroCostDL4Mic notebooks, model checkpoints are automatically saved, in Google Drive, during training which allows the users to continue training later from such a checkpoint (see Supplementary Note 4 for discussion on transfer learning).

Log-out if idle. Google Colab may disconnect significantly earlier than after 12 hours if it detects an interruption of user interaction or network training. Usually, this time-out happens after 30 to 90 minutes of idleness in our hands, i.e. code not running or lack of user interaction with the Google Colab interface. When the log-out occurs, local variables, installed packages and data stored outside any mounted drive are deleted. Hence, if the log-out occurs before training a model, cells setting up parameters for training such as paths or hyperparameters may have to be reset before training. With all of our notebooks, the models are automatically saved in Google Drive upon training completion, meaning that long training sessions do not have to be attended.

5.4. Inconsistent GPU access

Google Colab does not guarantee access to a GPU, as the number of current users may be larger than the number of available devices. It is not clear how access to a GPU is regulated, but it may be determined by traffic to the Google Colab servers. If no GPUs are available, Colab will offer to run the notebook using either a TPU, CPU (without acceleration), or as a local runtime (i.e. on the machine of the user). It should be noted though that these alternatives can be significantly slower than Google Colab GPU access. However, GPU access usually becomes available again from within a few hours to a day.

5.5. GPU type

Google Colab uses different GPUs that currently include NVIDIA K80, T4, P4, and P100 (as of October 2020). The user cannot decide which GPU will be available when using the notebook. According to the Google Colab (https://research.google.com/colaboratory/faq.html) FAQ, this is due to limitations in the provision of a free service to users which makes certain types of GPU unavailable at the time a notebook is used. In practice, this does not affect the performance of the models trained with ZeroCostDL4Mic. However, it will affect the speed at which networks can be trained and used. Therefore, users might encounter variability in training times as a consequence. To find out which type of GPU Google Colab is using, the user can play the first cell in each notebook which will give information on GPU access and type.

5.6. How to best handle Google Colab resources

Several steps can be taken by users of ZeroCostDL4Mic to optimise the usage of Google Colab's resources. Regarding the 12h maximum training time, we encourage users to change the number of epochs and training parameters so that the training takes less than 12h. This should be possible with all of the networks we provide. Nevertheless, we also provide the option to continue the training from a saved training checkpoint in case of a time-out. This allows the user to start from a pre-trained model and accumulate many rounds of steps (see Supplementary Note 4 about transfer learning).

For the log-out if idle issue, Google Colab cells can be played all at once (or a subset at once). In this case, the activated cells will run one after the other. This can be useful to ensure that the runtime does not disconnect until the completion of all the analytical steps and the user data saved in Google Drive.

ZeroCostDL4Mic is aimed to be an entry point to learn about DL methods where users can quickly train networks with their data. While ZeroCostDL4Mic, associated with the Google Colab platform, is entirely free to use, the resources available can be easily extended with small financial investments. For instance, the free 15 GB Google Drive storage space can be increased by purchasing more Google Drive storage from Google. In addition, Google is rolling out a Google Colab Pro (https://colab.research.google.com/signup#) version that offers faster GPUs, longer runtimes and access to more RAM. If these intermediate options are still not sufficient, ZeroCostDL4Mic notebooks can also be adapted to run on the user's own computer by connecting Google Colab to a local runtime (https://research.google.com/colaboratory/local-runtimes.html). This option allows the user to access their local files and resources (local GPU) directly from the Google Colab notebooks. This, of course, requires the users to invest in a powerful workstation.

5.7. Data privacy and ZeroCostDL4Mic

It is important to note that using ZeroCostDL4Mic may not always be suitable for the analysis of confidential data as the images need to be uploaded to Google Drive prior to analysis. Therefore, we advise users to read the general conditions of using a Google Drive account https://www.google.com/drive/terms-of-service/ before using our notebooks in case concerns about data protection exist. Importantly, Google claims no ownership rights over content stored in Google Drive, and the use of Google Colab is not different. In our hands, removal, editing, or organisation of files have not occurred when developing and testing ZeroCostDL4Mic. According to Google Colab terms of use, file modifications become more likely if Google's terms are breached (https://support.google.com/docs/answer/148505) or if the user has given specific permission for files to be edited.

To ensure data safety, we recommend our users to upload images without their associated metadata (all the information that details what the images actually are). If this simple precaution is taken, we do not foresee that data privacy issues would affect most microscopy image analysis needs, as images without their associated metadata are virtually worthless. It is important to note that users can also upload only the dataset required to train DL networks and perform the prediction locally to alleviate the issues with storing large amounts of sensitive data in the cloud.

Supplementary Note 6: Using ZeroCostDL4Mic in alternative cloud-computing platforms.

Although we developed our platform around Google Colab's online computational resources, the flexibility of use of the Jupyter notebooks allows us to port the analysis to any cloud solutions accepting Jupyter notebooks. To demonstrate that users can migrate the notebooks to other environments, for example, to exploit alternative GPUs to those available in Colab, or if the free service of colab should be discontinued, we tested two of our notebooks in two alternative resources. As an example for another free service providing GPU access, we imported our Deep-STORM notebook into the Deepnote online platform (https://deepnote.com) (Supplementary Fig. 4a-d). Deepnote provides online resources: Intel Haswell 2vCPU and ~5GB RAM for free for an unlimited amount of time (as of October 2020) and provides additional RAM and access to GPU for a fee (prices available upon request to Deepnote).

Here, we performed installation, training, quality control and prediction on CPU using test data provided on Deep-STORM GitHub developer's page (https://github.com/EliasNehme/Deep-STORM) without any modifications of the notebook. The only additional step required here was the pre-installation of 5 Python packages (TensorFlow, scikit-image, astropy, tqdm and numba) which could be simply done by editing the "requirements.txt" file in Deepnote (see Deepnote documentation: https://docs.deepnote.com/environment/custom-initialization for details). Supplementary Figure 4 shows a set of screenshots highlighting the interface and the different steps being carried out within Deepnote.

We explored the use of another cloud-based service, this time while incurring a financial charge. Here, we chose FloydHub, an online platform which provides access to fast GPUs, such as Tesla K80 and Tesla V100 and an easy to use interface (Supplementary Fig. 4e-h). Importantly, FloydHub runs with significantly more powerful CPU, more runtime RAM (up to 61GB), disk space (200GB) and runtime limits of up to 7 days which would allow more data to be loaded during training and longer training times than is possible in Google Colab.

To use ZeroCostDL4Mic notebooks in FloydHub, we created a new project and a new workspace. Here, FloydHub provides the option to import a repository from GitHub. Using this option and importing the ZeroCostDL4Mic repository from https://github.com/HenriquesLab/ZeroCostDL4Mic, makes all the notebooks immediately available on the FloydHub workspace. To use a notebook, the user needs to select which environment the notebook should run in. Here, we demonstrated the use of the StarDist 2D notebook.

Next, we chose a machine, which can be a CPU or GPU, depending on the requirements for training. FloydHub charges the user for GPU use by the hour (1.20 USD per hour).

Next, we downloaded the StarDist 2D network from this project's zenodo dataset repository (https://zenodo.org/record/3715492#.X9tojtj7TIU). This is done by opening a terminal (bash) instance in FloydHub and typing the following command to download the dataset:

*wget zenodo.org/record/**dataset_ID_number**/files/**dataset_name**.zip*

where **dataset_ID_number** is the unique 7-digit zenodo ID number of the dataset (also in the URL) and **dataset_name** is the name of the folder containing the dataset which in this case was 'Stardist_v2'. The user can then unzip the folder by typing:

*unzip **dataset_name**.zip*

Users can also upload their own data into FloydHub by following the instructions found here (https://docs.floydhub.com/guides/create_and_upload_dataset/). After these steps, the GitHub repository and the dataset folder are available in the created workspace. The StarDist 2D notebook can be opened from the left-hand menu and displays the same interface as in colab, with the exception of showing the code cells permanently. Three minor modifications had to be made to use the StarDist 2D notebook:

- comment out the *%tensorflow_version 1.x* magic command in cell 2 by prepending #
- the *from __future__ import ...* command was copied to the top of cell 2
- astropy needed to be installed before import by adding *!pip install astropy* to cell 2

These changes were sufficient to use the notebook for training, quality control and inference. Further changes to the notebook simplify the experience of use on this platform, e.g. deleting the string '/content' in pathnames or replacing it with '/floyd/home' where it occurs in the notebook. This is necessary because the 'content' folder is specific to Google Colab which will result in an error when using the notebook in FloydHub. Additionally, copying paths from the left-hand menu omits the root folder '/floyd/' which we needed to add manually when pasting a dataset or model path into the notebook.

These changes were made after running each cell and using any error messages to adapt the notebook to the new platform. Similar minor changes would likely be necessary to use the other notebooks. However, as we demonstrated, these changes could be made extremely quickly and the notebook could be used within minutes of creating a FloydHub account.

Supplementary Tables

Network	Data Type	# of files	Image Dimensions	Image Type	Comments
CARE (2D)	Training – Low SNR images	22	1024x1024	SIM fluo (MIP from 3D stack) 32-bit TIFF	This paper (Filopodia dataset - Maximum projection), and here
CARE (2D)	Training – High SNR images	22	1024x1024	SIM fluo (MIP from 3D stack) 32-bit TIFF	This paper (Filopodia dataset - Maximum projection), and here
CARE (2D)	Test – Low SNR images	2	1024x1024	SIM fluo (MIP from 3D stack) 32-bit TIFF	This paper (Filopodia dataset - Maximum projection), and here
CARE (2D)	Test – High SNR images	2	1024x1024	SIM fluo (MIP from 3D stack) 32-bit TIFF	This paper (Filopodia dataset - Maximum projection), and here
CARE (3D)	Training – Low SNR images	22	1024x1024x33	SIM fluo 32-bit TIF	This paper (Filopodia dataset 3D - stack), and here
CARE (3D)	Training – High SNR images	22	1024x1024x33	SIM fluo 32-bit TIF	This paper (Filopodia dataset 3D - stack), and here
CARE (3D)	Test – Low SNR images	2	1024x1024x33	SIM fluo 32-bit TIF	This paper (Filopodia dataset 3D - stack), and here
CARE (3D)	Test – High SNR images	2	1024x1024x33	SIM fluo 32-bit TIF	This paper (Filopodia dataset 3D - stack), and here
U-Net (2D)	Training - Images	28	512x512	EM 8-bit TIFF	ISBI or here
U-Net (2D)	Training - Masks	28	512x512	Binary 8-bit TIFF	ISBI or here
U-Net (2D)	Test - Images	2	512x512	EM 8-bit TIFF	ISBI or here
U-Net (2D)	Test - Masks	2	512x512	Binary 8-bit TIFF	ISBI or here
U-Net (3D)	Training/Test - Images	165	1024x768	EM 8-bit TIFF	here
U-Net (3D)	Training/Test - Masks	165	1024x768	Binary TIFF	here
Label-free prediction (fnet)	Training - Brightfield	92	512x512x32	Bright-field confocal 8-bit TIF	This paper, and here
Label-free prediction (fnet)	Training – Fluo (mitochondrial marker)	92	512x512x32	Fluo confocal 8-bit TIF	This paper, and here
Label-free prediction (fnet)	Test - Brightfield	8	512x512x32	Bright-field confocal 8-bit TIF	This paper, and here
Label-free prediction (fnet)	Test – Fluo (mitochondrial marker)	8	512x512x32	Fluo confocal 8-bit TIF	This paper, and here
Stardist (2D)	Training - Images	45	1024x1024	Fluo 16-bit TIF	This paper, and here
Stardist (2D)	Training - Masks	45	1024x1024	Object-labelled 8-bit TIF	This paper, and here
Stardist (2D)	Test - Images	2	1024x1024	Fluo 16-bit TIF	This paper, and here
Stardist (2D)	Test - Masks	2	1024x1024	Object labelled 8-bit TIF	This paper, and here
Stardist (2D)	Test - Stacks	4	1024x1024x86	Fluo 16-bit TIF	This paper, and here
Stardist (3D)	Training - Images	27	128x128x64	Fluo 16-bit TIF	Martin Weigert et al , 2020 and here
Stardist (3D)	Training - Masks	27	128x128x64	Fluo 16-bit TIF	Martin Weigert et al, 2020 and here
Stardist (3D)	Test - Images	3	128x128x64	Fluo 16-bit TIF	Martin Weigert et al, 2020 and here
Stardist (3D)	Test - Images	3	128x128x64	Fluo 16-bit TIF	Martin Weigert et al, 2020 and here
Deep-STORM	Training - Simulated SMLM data	2	64x64	16-bits/32-bits TIFF	This paper and here
Deep-STORM	Training - Ground truth localizations	2	n.a.	.csv file	This paper and here
Deep-STORM	Example dataset - Experimental SMLM data	2	256x256	16-bits/32-bits TIFF	This paper and here
CycleGAN	Training - Spinning Disk Images	164	1280x1280	8-bit PNG	This paper and here
CycleGAN	Training - (unpaired) Fluctuation based super resolution Images	164	1280x1280	8-bit PNG	This paper and here
CycleGAN	Test - Spinning Disk Images	4	1280x1280	8-bit PNG	This paper and here
CycleGAN	Test - Fluctuation based super resolution Images	4	1280x1280	8-bit PNG	This paper and here
pix2pix	Training - LifeAct Spinning disk Images	1748	1024x1024	8-bit PNG	This paper and here
pix2pix	Training - SiR DNA Spinning Disk Images	1748	1024x1024	8-bit PNG	This paper and here
pix2pix	Test - LifeAct Spinning disk Images	5	1024x1024	8-bit PNG	This paper and here
pix2pix	Test - SiR DNA Spinning Disk Images	5	1024x1024	8-bit PNG	This paper and here
YOLOv2	Training - Brightfield Images	30	1040x1380	8-bit PNG	This paper and here
YOLOv2	Training - Hand-annotations	30	n.a.	xml (Pascal VOC - format)	This paper and here
YOLOv2	Test - Brightfield Images	3	1040x1380	8-bit PNG	This paper and here
YOLOv2	Test - Hand-annotations	3	n.a.	xml (Pascal VOC - format)	This paper and here
N2V (2D)	Training - Images	1	512x512	Fluo 16-bit TIFF	Stubb et al 2020, and here
N2V (2D)	Test - Images	22	512x512	Fluo 16-bit TIFF	Stubb et al, 2020 and here
N2V (3D)	Training - Images	1 (Actin) +1 (Fibronectin)	512x512x13	Fluo 16-bit TIFF	Kaukonen et al, 2017 (Actin and fibronectin datasets), and here
N2V (3D)	Test - Images	48 (Actin) +48 (Fibronectin)	512x512x13	Fluo 16-bit TIFF	Kaukonen et al, 2017 (Actin and fibronectin datasets), and here

Supplementary Table 1: Overview of the available datasets used for training the networks. For all networks relying on supervised learning, the test datasets consist of the last two files generated for training. These were set aside for testing and are not part of the training dataset.

Network Name	# of epochs	# of steps	Batch size	Image dimensions	Bit-depth	# of images	# of patches, patch size, patch height	GPU type	Time for training (using these settings)
CARE (2D)	50	31	64	1024x1024	32-bit	22	100, 80	Tesla P100-PCIE-16GB	3 min
CARE (3D)	50	62	64	1024x1024x33	32-bit	22	200, 80, 8	Tesla P4	90 min
U-Net (2D)	200	6	4	1024x1024	8-bit	28	4, 512x512	Tesla P100-PCIE-16GB	8 min
U-Net (3D)	75	396	1	1024x768	8-bit	165	132, 256x256, 16	Tesla P100-PCIE-16GB	575 min
Label-free prediction (fnet)	n.a.	50000	4	512x512x32	8-bit	92	64, 64, 32	Tesla P4	8h20min
StarDist (2D)	200	37	2	1024x1024	16-bit	72	1, 1024x1024 .	Tesla K80	170 min
StarDist (3D)	400	14	2	128x128x64	16-bit	27	1, 128x128x64	Tesla P4	400 min
Deep-STORM	100	313	16	64x64	32-bit	20 (frames)	500, 26x26	Tesla P100-PCIE-16GB	50min
cycleGAN	200	n.a.	1	1280x1280	RGB	164	512x512	Tesla P100-PCIE-16GB	8h54min
pix2pix	200	n.a.	1	1024x1024	RGB	1748	512x512	Tesla P100-PCIE-16GB	9h
YOLOv2	40	30	8	1040x1380	8-bit	30	1, 1040x1380	Tesla P100-PCIE-16GB	45min
N2V (2D)	100	61	128	512x512	16-bit	22	392, 64	Tesla T4	17 min
N2V (3D)	100	133	128	512x512x13	16-bit	48	392, 64, 8	Tesla P100-PCIE-16GB	240 min

Supplementary Table 2: Hyperparameters used to train the networks, GPU types allocated and corresponding training times. Some hyperparameters are hard-coded in the networks or calculated from the other parameters. n.a.: not applicable.

Network Name	Install time	Image dimensions	Bit-depth	of images	Patch size	Default augmentation	Validation split	Total number of training patches	Tesla P100	Tesla T4	CPU (if possible)
CARE (2D)	25s	1024x1024	32-bit	22	80x80	None	0.1	1980	4s per EPOCH	6s per EPOCH	823s per EPOCH
CARE (3D)	25s	1024x1024x33	32-bit	22	80x80x8	None	0.1	3960	47s per EPOCH	102s per EPOCH	5134s per EPOCH
Unet (2D)	10s	512x512	8-bit	28	512x512	1x	0.1	26	2s per EPOCH	4s per EPOCH	415s per EPOCH
Unet (3D)	10s	1024x768x165	8-bit	1	256x256x16	4x	0.2	396	842s per EPOCH	932s per EPOCH	GPU required
Label-free prediction (fnet)	53s	512x512x32	8-bit	92	64x64x32	None	0.3	variable (depends on number of steps)	0.52s per STEP	0.54s per STEP	GPU required
StarDist (2D)	1 min	1024x1024	16-bit	72	1024x1024	None	0.1	64	42s per EPOCH	45s per EPOCH	881s per EPOCH
StarDist (3D)	1 min	128x128x64	16-bit	27	128x128x64	None	0.1	24	60s per EPOCH	65s per EPOCH	2360s per EPOCH
Deep-STORM	10s	64x64	32-bit	20	26x26	None	0.3	7000	31s per EPOCH	61s per EPOCH	2620s per EPOCH
cycleGAN	20s	1280x1280	RGB	164	512x512	x2	n.a.	1 per image	178s per EPOCH	315s per EPOCH	GPU required
pix2pix	15s	1024x1024	RGB	1748	512x512	None	n.a.	1 per image	168s per EPOCH	265s per EPOCH	GPU required
YOLOv2	17s	1389x1041	8-bit	30	n.a.	8x	0.1	n.a.	100s per EPOCH	102s per EPOCH	2386s per EPOCH
N2V (2D)	25s	512x512	16-bit	22	64x64	8x	0.1	9012	7s per EPOCH	11s per EPOCH	41s per EPOCH
N2V (3D)	25s	512x512x13	16-bit	1	64x64x4	8x	0.1	1383	7s per EPOCH	15s per EPOCH	641s per EPOCH

Supplementary Table 3: Notebooks and training times with respect to datasets and types of processing units (GPU and CPU if applicable) in the notebooks. n.a.: not applicable.

Network Name	Image dimensions	Bit-depth	Tesla P100	Tesla T4	CPU (if possible)
CARE (2D)	1024x1024	32-bit	0.6 s/frame	0.6 s/frame	13.7 s/frame
CARE (3D)	1024x1024x33	32-bit	8.3 s/stack	16s/stack	183.7s/stacks
Unet (2D)	512x512	8-bit	0.7 s/frame	1 s/frame	5 s/frame
Unet (3D)	1024x768x165	8-bit	47s/stack	69s/stack	2091 s/stack
Label-free prediction (fnet)	512x512x32	8-bit	9.1 s/stack	11.1 s/stack	GPU only
StarDist (2D)	1024x1024	16-bit	1.2 s/frame	1.7 s/frame	4.3 s/frame
StarDist (3D)	128x128x64	16-bit	5.9 s/stack	5.9 s/stack	25 s/stack
Deep-STORM	256x256	32-bit	0.2 s/frame	0.6 s/frame	6.4 s/frame
cycleGAN	1280x1280	RGB	2 s/frame	3 s/frame	GPU only
pix2pix	1024x1024	RGB	1.1 s/frame	1.9 s/frame	GPU only
YOLOv2	1389x1041	8-bit	CPU only	CPU only	9.3s/frame
N2V (2D)	512x512	16-bit	0.5 s/frame	1.9 s/frame	2.6 s/frame
N2V (3D)	512x512x13	16-bit	1.7 s/stack	1.9s/stack	25 s/stack

Supplementary Table 4: Notebooks and inference times with respect to datasets and types of processing units (GPU and CPU if applicable) in the notebooks. n.a.: not applicable.

Network	Image dimensions	Bit-depth	tested Patch size	Default augmentation	Number of patches	What is limiting?	Max number of training images with default parameters	Description of the breaking point
CARE (2D)	1024x1024	32-bit	80x80	None	100 per image	Runtime RAM	1000 paired images	all training patches need to fit in the runtime RAM
CARE (3D)	1024x1024x33	32-bit	80x80x8	None	200 per Stack	Runtime RAM	100 paired stacks	all training patches need to fit in the runtime RAM
Unet (2D)	512x512	8-bit	512x512	1x	1 per image	GDrive disk space	30,000 paired images	All training images need to fit on Google Drive disk space (15GB for free service)
Unet (3D)	1024x768x165	8-bit	256x256x16	4x	396	GDrive disk space	9,500	All training images must fit in GDrive disk space.
Label-free prediction (fnet)	512x512x32	8-bit	64x64x32	None	variable	Runtime RAM	142 paired stacks	all training stacks need to fit in the runtime RAM
StarDist (2D)	1024x1024	16-bit	1024x1024	None	1 per image	Runtime RAM	360 paired images	all training patches need to fit in the runtime RAM
StarDist (3D)	128x128x64	16-bit	128x128x64	None	1 per Stack	Runtime RAM	90 paired stacks	all training patches need to fit in the runtime RAM
Deep-STORM	256x256	32-bit	26x26	None	10000 total	Runtime RAM	10000 patches	all training patches need to fit in the runtime RAM
cycleGAN	1280x1280	RGB	512x512	x2	1 per image	GDrive disk space	5000 images (training source + training target)	Trained models take a lot of space (around 10GB for 200 EPOCH)
pix2pix	1024x1024	RGB	512x512	None	1 per image	GDrive disk space	4000 paired images	Trained models are large (around 10GB for 200 EPOCH)
YOLOv2	1389x1041	8-bit	n.a.	x8	n.a.	GDrive disk space	20000 images+annotations	All training images need to fit on Google Drive disk space (15GB for free service)
N2V (2D)	512x512	16-bit	64x64	x8	512 per image	Runtime RAM	170 images (1360 with no augmentation)	all training patches need to fit in the runtime RAM
N2V (3D)	512x512x13	16-bit	64x64x4	x8	1536 per stack	Runtime RAM	15 stacks (120 with no augmentation)	all training patches need to fit in the runtime RAM

Supplementary Table 5: Notebooks and notebook breaking points for training with respect to datasets. n.a.: not applicable. Breaking points were determined empirically by performing training with an increasing number of training images until the Colab session crashed (often due to exceeding the RAM limit, as highlighted in the table). Where this breaking point was not reached, the limitation of the training dataset size is determined by the maximum space available on the Google Drive (corresponding to the 15GB of data available in the session). In this case, values for the dataset size are estimated based on the file size in the given dataset.

Network	Image size tested	Bit-depth	Maximum number of images per runtime	Description of the issue
CARE (2D)	1024x1024	32-bit	1700 images	GDrive disk space
CARE (3D)	1024x1024x33	32-bit	50 stacks	GDrive disk space
Unet (2D)	512x512	8-bit	30,000 images	GDrive disk space
Unet (3D)	1024x768x165	8-bit	23 stacks	GDrive disk space
Label-free prediction (fnet)	512x512x32	8-bit	200 stacks	GDrive disk space
StarDist 2D	1024x1024	16-bit	2000 images	GDrive disk space
StarDist 3D	128x128x64	16-bit	2300 stacks	GDrive disk space
Deep-STORM	256x256	32-bit	50,000 frames	GDrive disk space
cycleGAN	1280x1280	RGB	3000 images	GDrive disk space
pix2pix	1024x1024	RGB	3000 images	GDrive disk space
YOLOv2	1389x1041	8-bit	7500 images	GDrive disk space
N2V 2D	512x512	16-bit	9000 images	GDrive disk space
N2V 3D	512x512x13	16-bit	700 stacks	GDrive disk space

Supplementary Table 6: Notebooks and notebook breaking points for inference with respect to datasets. n.a.: not applicable. Breaking points were determined by running the inference cell in the notebooks on an increasing number of files. Since inference is usually done at a fixed batch size it is usually not limited by RAM but by maximum memory of the Google Drive. In this case, values for the dataset size are estimates based on the file size in the given dataset

Bibliography

1. Gómez-de-Mariscal, E. *et al. DeepImageJ: A user-friendly plugin to run deep learning models in ImageJ*. http://biorxiv.org/lookup/doi/10.1101/799270 (2019) doi:10.1101/799270.
2. Schmidt, U., Weigert, M., Broaddus, C. & Myers, G. Cell Detection with Star-Convex Polygons. in *Medical Image Computing and Computer Assisted Intervention – MICCAI 2018* (eds. Frangi, A. F., Schnabel, J. A., Davatzikos, C., Alberola-López, C. & Fichtinger, G.) vol. 11071 265–273 (Springer International Publishing, 2018).
3. Ouyang, W., Mueller, F., Hjelmare, M., Lundberg, E. & Zimmer, C. ImJoy: an open-source computational platform for the deep learning era. *ArXiv190513105 Cs Q-Bio Stat* (2019).
4. Berg, S. *et al.* ilastik: interactive machine learning for (bio)image analysis. *Nat. Methods* **16**, 1226–1232 (2019).
5. Legant, W. R. *et al.* High-density three-dimensional localization microscopy across large volumes. *Nat. Methods* **13**, 359–365 (2016).
6. Jin, L. *et al.* Deep learning enables structured illumination microscopy with low light levels and enhanced speed. *Nat. Commun.* **11**, 1934 (2020).
7. Ounkomol, C., Seshamani, S., Maleckar, M. M., Collman, F. & Johnson, G. R. Label-free prediction of three-dimensional fluorescence images from transmitted-light microscopy. *Nat. Methods* **15**, 917–920 (2018).
8. Weigert, M. *et al.* Content-aware image restoration: pushing the limits of fluorescence microscopy. *Nat. Methods* **15**, 1090–1097 (2018).
9. Belthangady, C. & Royer, L. A. Applications, promises, and pitfalls of deep learning for fluorescence image reconstruction. *Nat. Methods* **16**, 1215–1225 (2019).
10. von Chamier, L., Laine, R. F. & Henriques, R. Artificial intelligence for microscopy: what you should know. *Biochem. Soc. Trans.* **47**, 1029–1040 (2019).
11. Moen, E. *et al.* Deep learning for cellular image analysis. *Nat. Methods* **16**, 1233–1246 (2019).
12. Antun, V., Renna, F., Poon, C., Adcock, B. & Hansen, A. C. On instabilities of deep learning in

image reconstruction and the potential costs of AI. *Proc. Natl. Acad. Sci.* 201907377 (2020) doi:10.1073/pnas.1907377117.

13. Wang, Z., Bovik, A. C., Sheikh, H. R. & Simoncelli, E. P. Image Quality Assessment: From Error Visibility to Structural Similarity. *IEEE Trans. Image Process.* **13**, 600–612 (2004).
14. Weigert, M., Schmidt, U., Haase, R., Sugawara, K. & Myers, G. Star-convex Polyhedra for 3D Object Detection and Segmentation in Microscopy. 8 (2020).
15. Everingham, M., Van Gool, L., Williams, C. K. I., Winn, J. & Zisserman, A. The Pascal Visual Object Classes (VOC) Challenge. *Int. J. Comput. Vis.* **88**, 303–338 (2010).
16. Girshick, R., Donahue, J., Darrell, T. & Malik, J. Region-Based Convolutional Networks for Accurate Object Detection and Segmentation. *IEEE Trans. Pattern Anal. Mach. Intell.* **38**, 142–158 (2016).
17. Boyd, K., Eng, K. H. & Page, C. D. Area under the Precision-Recall Curve: Point Estimates and Confidence Intervals. in *Advanced Information Systems Engineering* (eds. Salinesi, C., Norrie, M. C. & Pastor, Ó.) vol. 7908 451–466 (Springer Berlin Heidelberg, 2013).
18. Everingham, M. & Winn, J. The PASCAL Visual Object Classes Challenge 2010 (VOC2010) Development Kit. 29.
19. Padilla, R., Netto, S. L. & da Silva, E. A. B. A Survey on Performance Metrics for Object-Detection Algorithms. in *2020 International Conference on Systems, Signals and Image Processing (IWSSIP)* 237–242 (IEEE, 2020). doi:10.1109/IWSSIP48289.2020.9145130.
20. Lobo, J. M., Jiménez-Valverde, A. & Real, R. AUC: a misleading measure of the performance of predictive distribution models. *Glob. Ecol. Biogeogr.* **17**, 145–151 (2008).
21. Van Rijsbergen, C. (1979). *Inf. Retr. 2nd Ed. Lond. Engl. Butterworths* (1979).
22. Caicedo, J. C. *et al.* Evaluation of Deep Learning Strategies for Nucleus Segmentation in Fluorescence Images. *Cytometry A* **95**, 952–965 (2019).
23. Moen, E. *et al. Accurate cell tracking and lineage construction in live-cell imaging experiments with deep learning*. http://biorxiv.org/lookup/doi/10.1101/803205 (2019) doi:10.1101/803205.

24. Kirillov, A., He, K., Girshick, R., Rother, C. & Dollár, P. Panoptic Segmentation. *ArXiv180100868 Cs* (2019).

25. Bloice, M. D., Roth, P. M. & Holzinger, A. Biomedical image augmentation using Augmentor. *Bioinformatics* **35**, 4522–4524 (2019).

26. Jung, A. *imgaug-doc* https://github.com/aleju/imgaug-doc.

27. Pan, S. J. & Yang, Q. A Survey on Transfer Learning. *IEEE Trans. Knowl. Data Eng.* **22**, 1345–1359 (2010).

28. Athanasiadis, I., Mousouliotis, P. & Petrou, L. A Framework of Transfer Learning in Object Detection for Embedded Systems. *ArXiv181104863 Cs* (2018).
