# Supplementary material for: Democratising deep learning for microscopy with ZeroCostDL4Mic
Source: Nat Commun. 2021 Apr 15;12:2276. doi: 10.1038/s41467-021-22518-0 (PMC8050272; doi:10.1038/s41467-021-22518-0)
Supplement: Supplementary file 13 — Reporting Summary [file 41467_2021_22518_MOESM13_ESM.pdf]

nature research

Corresponding author(s): Jacquemet, Henriques

Last updated by author(s): Feb 18, 2021

Reporting Summary

Nature Research wishes to improve the reproducibility of the work that we publish. This form provides structure for consistency and transparency in reporting. For further information on Nature Research policies, see our Editorial Policies and the Editorial Policy Checklist.

Statistics

For all statistical analyses, confirm that the following items are present in the figure legend, table legend, main text, or Methods section.

n/a	Confirmed	
☐	☒	The exact sample size (*n*) for each experimental group/condition, given as a discrete number and unit of measurement
☐	☒	A statement on whether measurements were taken from distinct samples or whether the same sample was measured repeatedly
☒	☐	The statistical test(s) used AND whether they are one- or two-sided<br>*Only common tests should be described solely by name; describe more complex techniques in the Methods section.*
☒	☐	A description of all covariates tested
☒	☐	A description of any assumptions or corrections, such as tests of normality and adjustment for multiple comparisons
☐	☒	A full description of the statistical parameters including central tendency (e.g. means) or other basic estimates (e.g. regression coefficient) AND variation (e.g. standard deviation) or associated estimates of uncertainty (e.g. confidence intervals)
☒	☐	For null hypothesis testing, the test statistic (e.g. *F*, *t*, *r*) with confidence intervals, effect sizes, degrees of freedom and *P* value noted<br>*Give P values as exact values whenever suitable.*
☒	☐	For Bayesian analysis, information on the choice of priors and Markov chain Monte Carlo settings
☒	☐	For hierarchical and complex designs, identification of the appropriate level for tests and full reporting of outcomes
☒	☐	Estimates of effect sizes (e.g. Cohen's *d*, Pearson's *r*), indicating how they were calculated

Our web collection on statistics for biologists contains articles on many of the points above.

Software and code

Policy information about availability of computer code

Data collection	Images were collected as indicated in the materiel and methods. All the instruments used are commercially available and were controlled using the software provided by the manufacturer. Software used to control the microscopes include: Zen blue (2.3 sp1), SlideBook 6, NIS-Elements (version 4.4) and SoftWorx (3.7).
Data analysis	Images used in the manuscript were processed with Fiji (2.1.0), ZeroCostDL4Mic (1.12.2) or Imaris (8.1.2) as indicated. Others software used include TrackMate (v6), motilitylab.net (no version available), ThunderSTORM (1.3), makesense.ai (1.7), and plotsofdata (1.05) .

For manuscripts utilizing custom algorithms or software that are central to the research but not yet described in published literature, software must be made available to editors and reviewers. We strongly encourage code deposition in a community repository (e.g. GitHub). See the Nature Research guidelines for submitting code & software for further information.

Data

Policy information about availability of data

All manuscripts must include a data availability statement. This statement should provide the following information, where applicable:

- Accession codes, unique identifiers, or web links for publicly available datasets
- A list of figures that have associated raw data
- A description of any restrictions on data availability

All our example datasets are available for download in Zenodo (Links also available in our GitHub page): CARE 3D (doi:10.5281/ZENODO.3713337),CARE 2D (doi:10.5281/ZENODO.3713330), Noise2Void 3D (doi:10.5281/ZENODO.3713326), Noise2Void 2D (doi:10.5281/ZENODO.3713315), Deep-STORM (doi:10.5281/ZENODO.3959089), CycleGAN (doi:10.5281/ZENODO.3941884), pix2pix (doi:10.5281/ZENODO.3941889), YOLOv2 (doi:10.5281/ZENODO.3941908), StarDist 2D (doi:10.5281/ZENODO.3715492), Label-free prediction fnet (doi:10.5281/ZENODO.3748967). The datasets used to train 2D Unet were originally published as part of

the ISBI 2012 segmentation challenge and were retrieved for this work from https://github.com/zhixuhao/unet. The dataset used for 3D segmentation in U-Net 3D is publicly available from the page of the École polytechnique fédérale de Lausanne (EPFL): https://www.epfl.ch/labs/cvlab/data/data-em/. In addition, a Source data file containing the raw data presented in Fig9c and Fig9e is provided with this paper.

Field-specific reporting

Please select the one below that is the best fit for your research. If you are not sure, read the appropriate sections before making your selection.

☒ Life sciences ☐ Behavioural & social sciences ☐ Ecological, evolutionary & environmental sciences

For a reference copy of the document with all sections, see nature.com/documents/nr-reporting-summary-flat.pdf

Life sciences study design

All studies must disclose on these points even when the disclosure is negative.

Sample size	No measure were taken to estimate the sample sizes. The sample size chosen represent typical imaging dataset generated while investigating biological phenomena. The sample size chosen were adequate as they were sufficient to train deep learning models of quality.
Data exclusions	No data were excluded.
Replication	Unless otherwise specified, all experiments were performed once. Here, we are assessing the performance of computer algorithms and not the variability of biological systems.
Randomization	The images used to train and test the deep learning models presented here were chosen randomly amount each dataset.
Blinding	Here data analysis was automatically performed and scored by computer algorithms, as the analyses are not affected by human bias, no blinding was necessary.

Reporting for specific materials, systems and methods

We require information from authors about some types of materials, experimental systems and methods used in many studies. Here, indicate whether each material, system or method listed is relevant to your study. If you are not sure if a list item applies to your research, read the appropriate section before selecting a response.

Materials & experimental systems

n/a	Involved in the study	
☐	☒	Antibodies
☐	☒	Eukaryotic cell lines
☒	☐	Palaeontology and archaeology
☒	☐	Animals and other organisms
☒	☐	Human research participants
☒	☐	Clinical data
☒	☐	Dual use research of concern

Methods

n/a	Involved in the study	
☒	☐	ChIP-seq
☒	☐	Flow cytometry
☒	☐	MRI-based neuroimaging

Antibodies

Antibodies used	TOM20 (sc-11415, Santa Cruz, USA); mouse-anti-β-tubulin primary antibody (ThermoFisher, catalogue number 32-2600); anti-tubulin antibody (clone 12G10, Developmental Studies Hybridoma Bank). Donkey-anti-rabbit-secondary antibody (Alexa Fluor 594 conjugated, A32754, Thermo Fisher, USA).
Validation	All primary antibodies used for immunofluorescence were validated by us using visual inspections and they all localized to the appropriate sub-cellular structures. These antibodies are all commercial and also well validated by the manufacturers.

Eukaryotic cell lines

Policy information about cell lines

Cell line source(s)	U2OS cells were purchased from DSMZ (Leibniz Institute DSMZ-German Collection of Microorganisms and Cell Cultures, Braunschweig DE, ACC 785). MDA-MB-231 cells were provided by ATCC. DCIS.COM cells were provided by J.F. Marshall (Barts Cancer Institute, Queen Mary University of London, London, England, UK). HeLa cells were provided by ECACC. U-251 glioma cells were provided by J. Ivaska (University of Turku, Turku, Finland). A2780 cells were a kind gift of P. Caswell (University of Manchester, Manchester, UK).
Authentication	Cell lines were not authenticated

Mycoplasma contamination	Cells lines used tested negative for Mycoplasma contamination
Commonly misidentified lines (See ICLAC register)	None
