# Supplementary material for: Democratising deep learning for microscopy with ZeroCostDL4Mic
Source: Nat Commun. 2021 Apr 15;12:2276. doi: 10.1038/s41467-021-22518-0 (PMC8050272; doi:10.1038/s41467-021-22518-0)
Supplement: Supplementary file 14 — Supplementary Software [file 41467_2021_22518_MOESM14_ESM.zip › Colab_notebooks/ZeroCostDL4Mic_UserManual_v1.3.pdf]

ZeroCostDL4Mic
User Manual

Henriques and Jacquemet labs

v1.3 – February 2021

Table of Contents

What is ZeroCostDL4Mic? 3

- Overview of the workflow 3
- Networks currently implemented 4
- Online resources 5

Preparing the data for ZeroCostDL4Mic 6

- Obtaining the dataset 6
- Uploading the dataset to Google Drive 6

Getting started with the notebook and initialising the Colab session 7

- Opening the notebook 7
- Initialising the Colab session 8
- Installing network components and related dependencies 9

Preparing for training 10

- Setting the main training parameters 10
- Data augmentation 11
- Transfer learning 12

Training the model and performing quality control 14

- Training 14
- Quality control on the trained model 15

Using the trained model to obtain predictions on new data 18

Final notes 20

References 21

A. What is ZeroCostDL4Mic?

ZeroCostDL4Mic is a toolbox for the training and implementation of common Deep Learning approaches to microscopy imaging (Figure 1). It exploits the ease-of-use and access to GPU provided by Google Colab. Training data can be uploaded to the Google Drive from where it can be used to train models using the provided Colab notebooks in a web-browser. Predictions on unseen data can then be performed within the same notebook, therefore not requiring any local hardware or software set-up.

This guide describes the workflow of a typical notebook run-through in a step-by-step fashion. You can also refer to this video for a complete walkthrough.

https://www.youtube.com/watch?v=GzD2gamVNHI&feature=youtu.be

A.1. Overview of the workflow

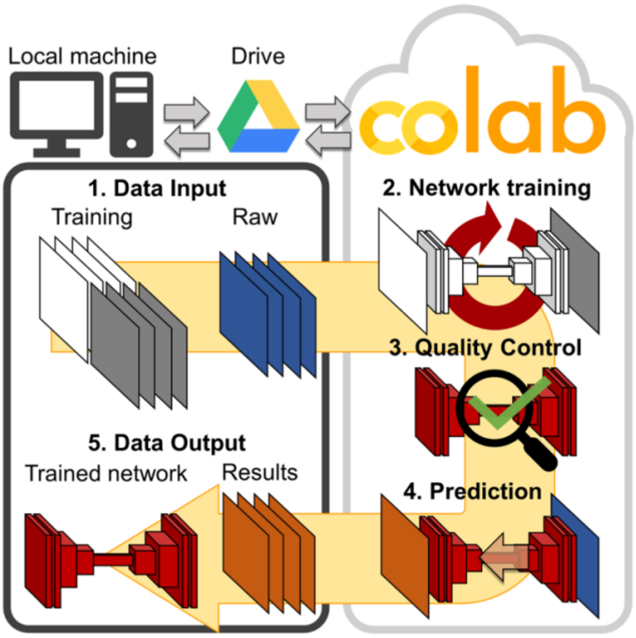

Figure 1: Workflow of ZeroCostDL4Mic. From data upload, execution of training, quality control and prediction on the cloud, to results and trained model download.

ZeroCostDL4Mic exploits online resources made available through Google Colab. **The general workflow comprises the following steps and is showcased in the following sections A-E**:

- i) Uploading data to Google Drive
- ii) Opening Colab Notebook online in Google Colab
- iii) Connecting the Google Drive to the current Colab session
- iv) Installation of network components dependencies
- v) Set training parameters (including transfer learning and data augmentation)
- vi) Run the training
- vii) Run Quality control on the trained model to ensure the validity of the trained model
- viii) Run predictions on unseen dataset
- ix) Download the trained network and the predictions on the unseen datasets

A.2. Networks currently implemented

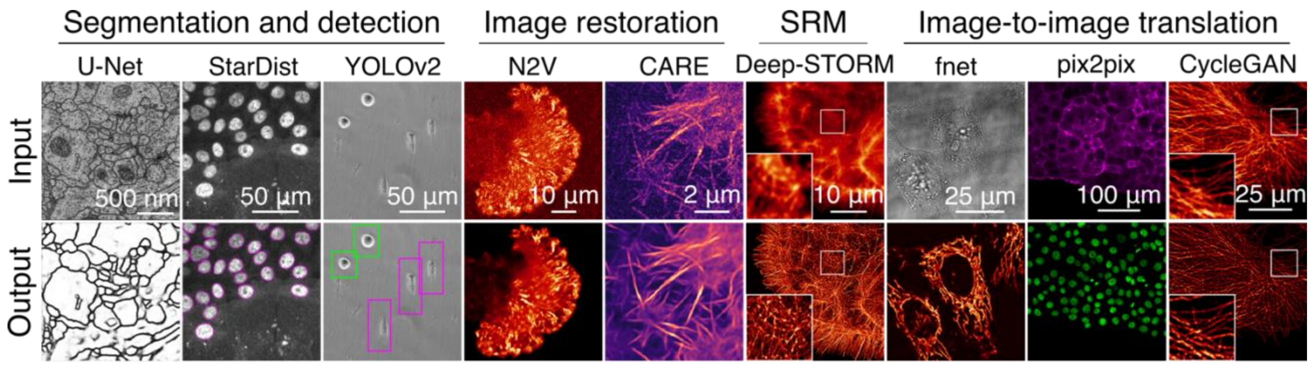

Figure 2: Networks and tasks currently implemented in ZeroCostDL4Mic. Segmentation and object detection, image restoration (including denoising), super-resolution microscopy (SRM) and image-to-image translation (including artificial labelling) are currently available.

- **U-Net (2D and 3D)**: for segmentation of bioimages, typically from electron microscopy data or bright-field imaging using supervised learning[1,2].
- **YOLOv2:** can classify objects and draw bounding boxes around detected objects. The original work is found here[3].
- **Noise2Void (2D and 3D)**: for denoising directly from the raw data, unsupervised so no need for paired training dataset[4].
- **StarDist (2D and 3D)**: for segmentation of cell nuclei in fluorescence images, specifically designed to detect round/oval objects using supervised learning[5,6].
- **CARE (2D and 3D)**: provides image restoration (denoising and resolution enhancement, among other features) using fully supervised learning[7].
- **Label-free prediction (fnet)**: predicts pseudo-fluorescence images from bright-field or EM data using supervised learning. The original work is found here[8].
- **pix2pix**: translates an image belonging to a specific image domain type into another image domain type. The original work is described here[9].
- **CycleGAN**: translates an image belonging to a specific image domain type into another image domain type without requiring the training dataset to be paired (unsupervised learning). The original work is described here[10].
- **Deep-STORM**: processes high-density data from single-molecule localization microscopy (SMLM) acquisitions. Supervised learning is performed on simulated single-molecule data. The original work is described here[11].

A.3. Online resources

Code development is a never-ending endeavour. We keep the framework alive by constantly developing new capabilities and adding new tasks and networks. Therefore, we make available many useful pieces of information via our GitHub page.

Below are a few links to specific online resources:

Google Colab

https://colab.research.google.com/notebooks/intro.ipynb

Link to our bioRxiv paper

https://www.biorxiv.org/content/10.1101/2020.03.20.000133v2

Wiki page on our GitHub repository

https://github.com/HenriquesLab/ZeroCostDL4Mic/wiki

Reporting bugs or issues

https://github.com/HenriquesLab/ZeroCostDL4Mic/issues

Page to the latest releases of the notebooks

https://github.com/HenriquesLab/ZeroCostDL4Mic/releases

Tips, tricks and FAQs

https://github.com/HenriquesLab/ZeroCostDL4Mic/wiki/Tips,-tricks-and-FAQs

Zenodo (dataset repository, use *ZeroCostDL4Mic* in the search bar)

https://zenodo.org

B. Preparing the data for ZeroCostDL4Mic

First, you should decide what task you want to perform using ZeroCostDL4Mic: we currently provide published networks that can perform image segmentation, object detection, denoising, restoration, artificial labelling and image-to-image translation. Please refer to our main Wiki page for info on the currently implemented notebooks. Users should also read our bioRxiv preprint[12] on the general framework to understand whether that is the right tool for the application they have in mind.

B.1. Obtaining the dataset

To start using the provided DL networks, you need some training datasets. You can either test the networks on the example training and test dataset that we provide or generate your own training dataset to be used on your own data. The different pages of our Wiki describe how to acquire the data you need in order to train the different networks.

If you decide to simply test the networks on our example training and test dataset, you can follow the different **Zenodo links that we provide** on our main Wiki page (Figure 3). You can download the whole dataset by clicking on the **Download** button on the Zenodo page. For U-Net, we do not provide a dataset ourselves but point towards an available dataset generated initially for the ISBI 2012 Segmentation challenge[13], see the U-Net page for details.

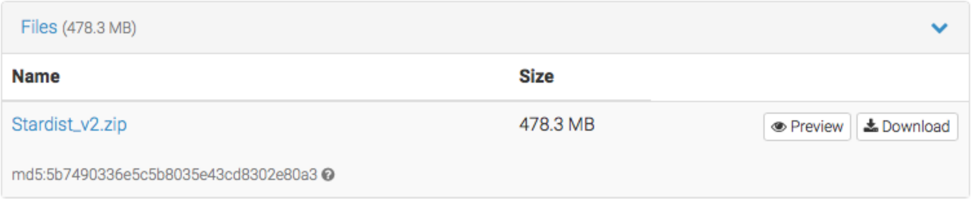

Figure 3: Obtaining our example training and test dataset: Zenodo download link, from https://zenodo.org/record/3715492#.Xo8bki2ZNQK

B.2. Uploading the dataset to Google Drive

For Google Colab to have access to these datasets, they need to be uploaded to your Google Drive.

You can do that simply by logging into your Google Drive account, clicking on 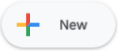

and then use the *Folder upload* option (Figure 4). **Please ensure that ALL the data is uploaded properly before proceeding further and that the data structure matches the requirements of the notebook to be used. You can find the requirements in section '0. Before getting started'.**

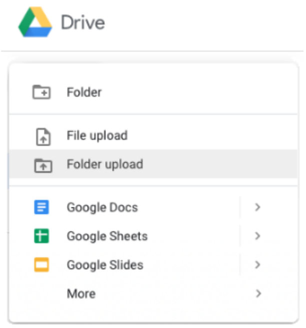

Figure 4: Uploading data to Google Drive.

C. Getting started with the notebook and initialising the Colab session

C.1. Opening the notebook

You can directly open the Colab notebook from our main Wiki page, by clicking on the corresponding **Open In Colab badge** Open in Colab . This will automatically open the notebook in Google Colab (Figure 5). Here, we have chosen to use the Dark mode of the notebook (white text on black background) for improved visibility.

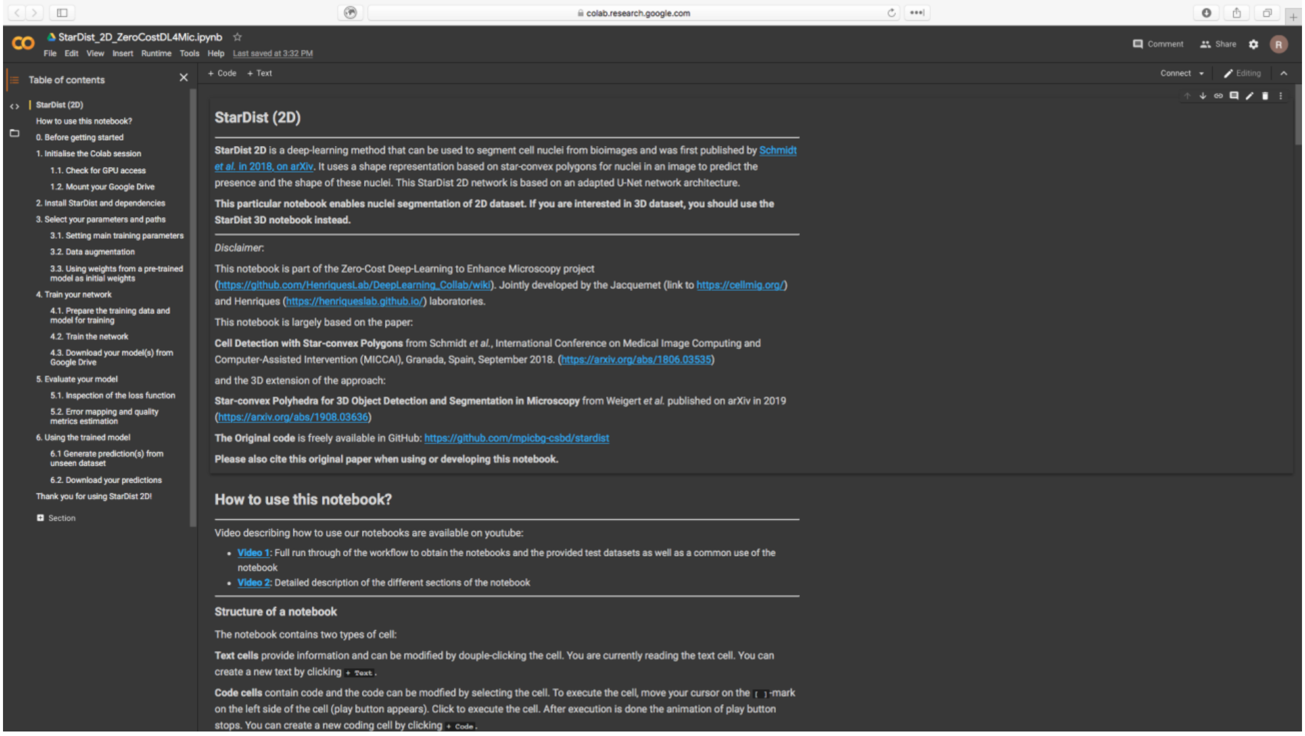

Figure 5: The ZeroCostDL4Mic notebook layout. The notebook shown here is that of StarDist (2D). The table of contents on the left highlights the different sections in the notebook.

On the left-hand side, you will find a **table of contents** that can be navigated through by clicking on the different items. It is important that you read the **0. Before getting started** section.

At that stage, you will not be able to make changes to the notebook unless you **save a copy of the notebook**. This can be easily done by going into *File* and *Save a copy in Drive…*. If you are signed into a Google Drive account, this will automatically save a copy of the notebook in your Google Drive in the Colab Notebooks folder (Figure 6).

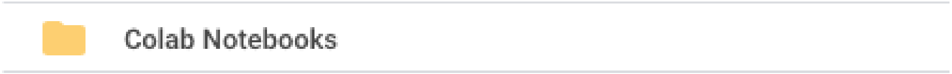

Figure 6: Colab Notebooks folder in Google Drive, where all copies of existing notebooks appear by default. The notebooks can be subsequently moved to other folders.

C.2. Initialising the Colab session

Now you're in the notebook, the first step is to check whether Google Colab will currently allow GPU access and, if so, what type. You can do this by running the **1.1 Check for GPU access** section (Figure 7). The GPU type is described on the last line of the Cell output. Here, we have been allocated a **Tesla K80**.

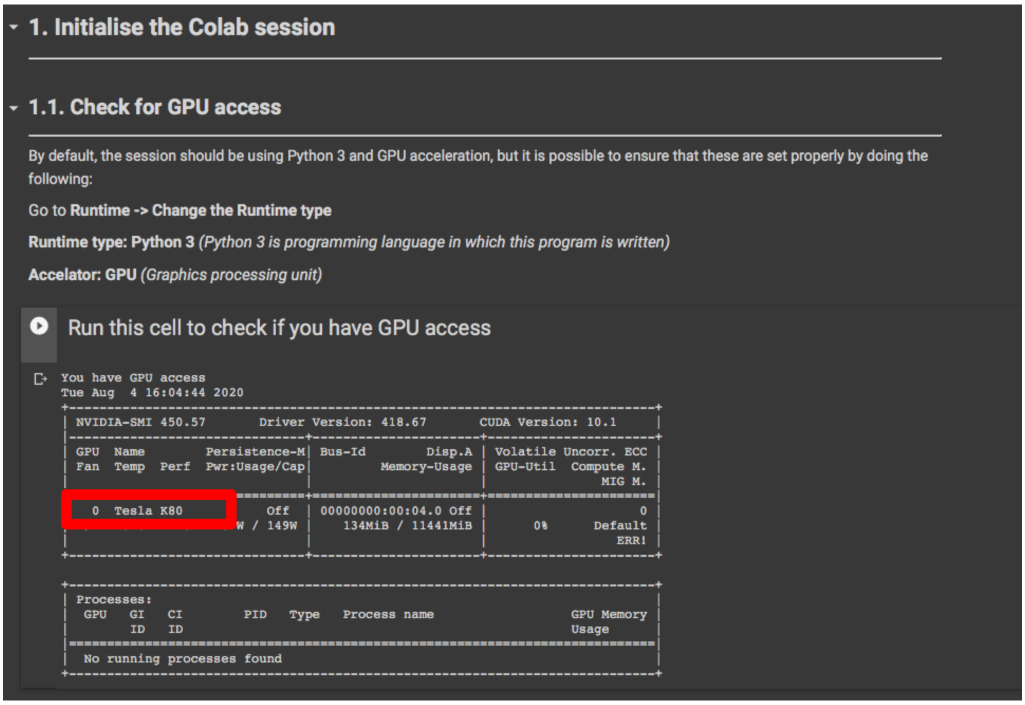

Figure 7: Checking GPU availability and type.

Then, you need to give Google Colab access to the data that you uploaded into your Google Drive. This step is called **Mounting the drive** and is executed in the **1.2 Mount your Google Drive** section (Figure 8). You will need to be logged in and provide an access code accessible via the provided link in the notebook.

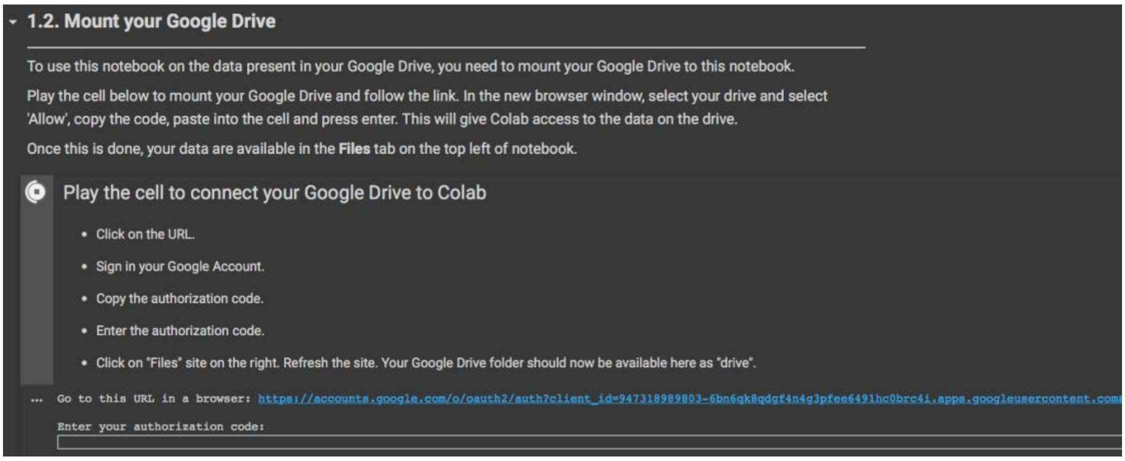

Figure 8: Mounting Google Drive to the Colab session. The link provided in the cell enables the authentication of the Google Drive and will provide the corresponding authorization code.

Once mounted, the drive and all its content are accessible via the dedicated tab on the left of the page (Figure 9).

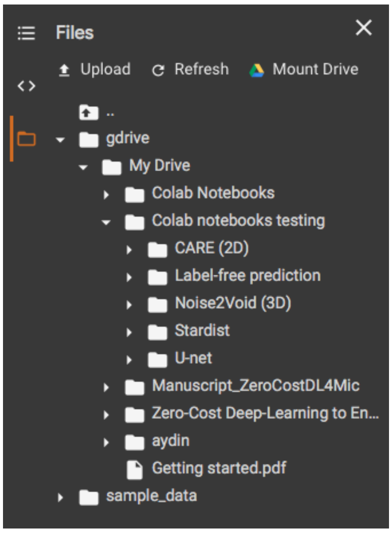

Figure 9: The File tab on the left of the notebook page gives access to the content of the mounted Google Drive.

C.3. Installing network components and related dependencies

Then, we are ready to install the network components and the required dependencies on the virtual machine made available to us by Google Colab. This is done by running the cell corresponding to the **section 2** of the notebook.

Important note: After running section 2, if you already have a trained model and wish to perform quality control on it, you can simply jump to **5. Evaluate your model** section. Similarly, if you only wish to run predictions on a new unseen dataset, you can jump directly to **6. Use the network** section.

D. Preparing for training

D.1. Setting the main training parameters

Section 3 of the notebook then provides critical information and requires important user input to prepare for the network training. The first part of section 3 highlights and describes the different parameters that the user will need to provide in order to prepare for training the model. This will include **providing the location (path, on the Google Drive) of the different parts of the training dataset** (source and target for fully supervised networks such as U-Net, StarDist, CARE and Label-free prediction, or only the source training dataset for unsupervised networks like Noise2Void). The other parts of the user input will be the training parameters, **e.g. number of epochs, number of steps, batch size**. If you are unsure about the meaning of these parameters, you can also refer to our Glossary page.

Some parameters are currently considered as advanced and can simply be left as their default values if the user wants to get started with a simple training session.

A typical section 3 user input will look like shown in Figure 10. **Do not forget to run this cell for the notebook to take your input into account**.

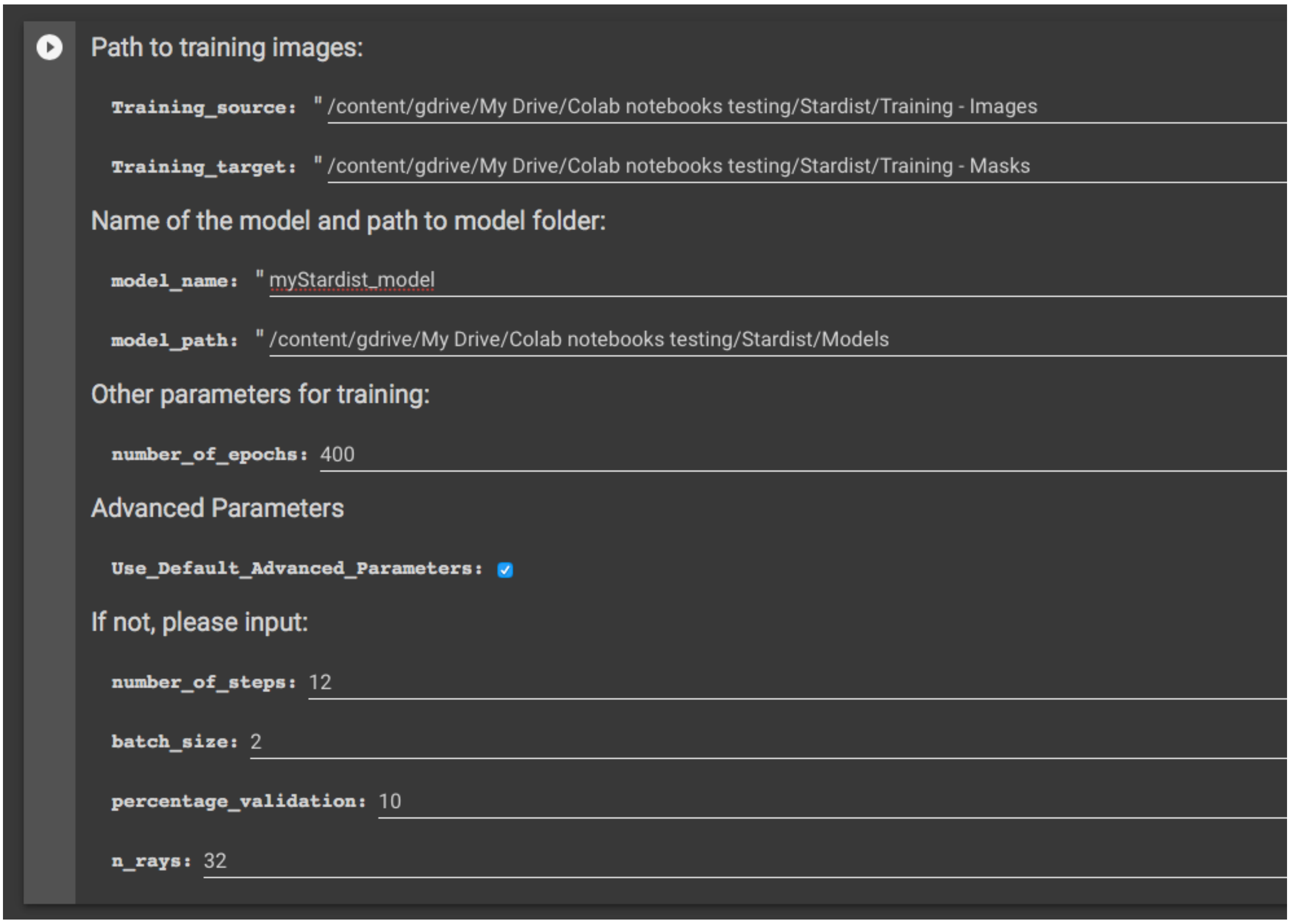

Figure 10: Setting the training parameters. Should be provided: paths to training dataset, name for the new model, path to where the new model will be created and saved, network-specific training parameters. Advanced parameters are also accessible.

For some networks, the notebook will highlight a randomly selected pair of source and target images from the training dataset for inspection that the data was uploaded and mounted properly (Figure 11).

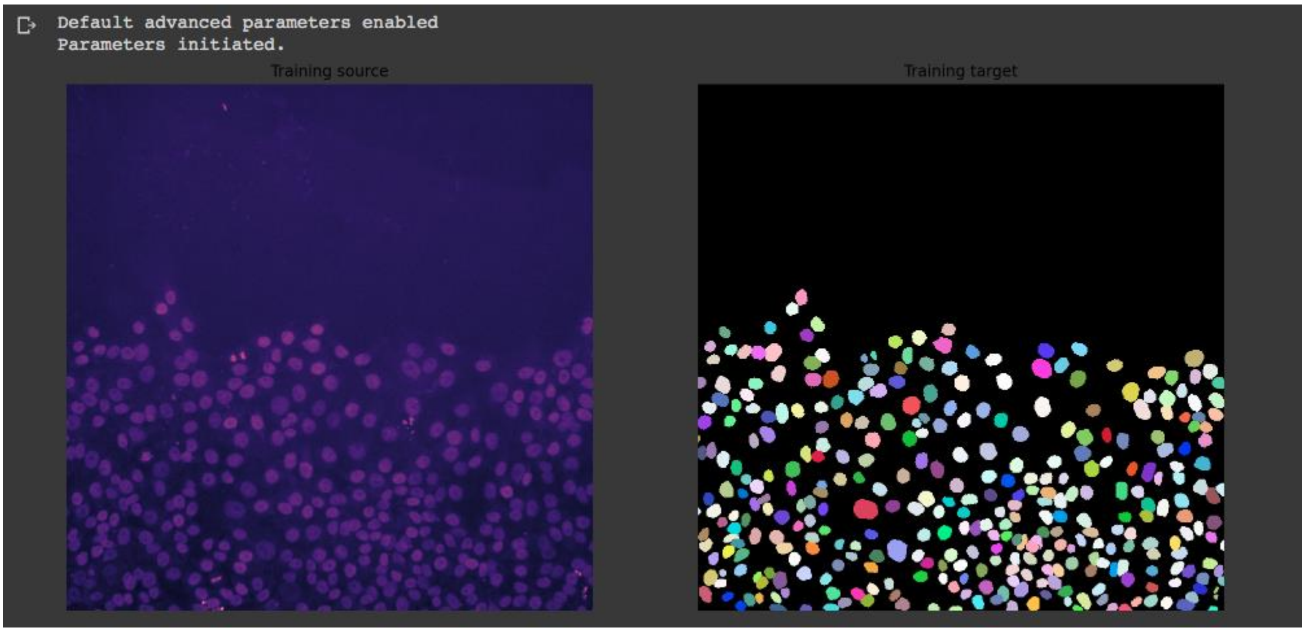

Figure 11: Display of an example training dataset pair for StarDist 2D. The left image shows the raw data, while the right image represents the nuclei masks. This dataset was taken from the example training dataset that we provide.

D.2. Data augmentation

The following section includes the option of performing **data augmentation** on the currently loaded training dataset. Data augmentation **artificially increases the size of the training dataset** by randomly applying spatial transformations to training image pairs (both source and target), therefore increasing the diversity of the data. The spatial transformations can be flipping with respect to the horizontal or vertical axes, rotations, zoom, shear, skew etc. The random aspect can be included in the choice of which transformations to apply and/or to what extent.

In Figure 12, we show an example of data augmentation notebook section (based on StarDist 2D notebook), highlighting the large choice of transformations that can be used. In particular, for StarDist 2D, **the Augmentor package**[14] **was used** to perform the augmentation.

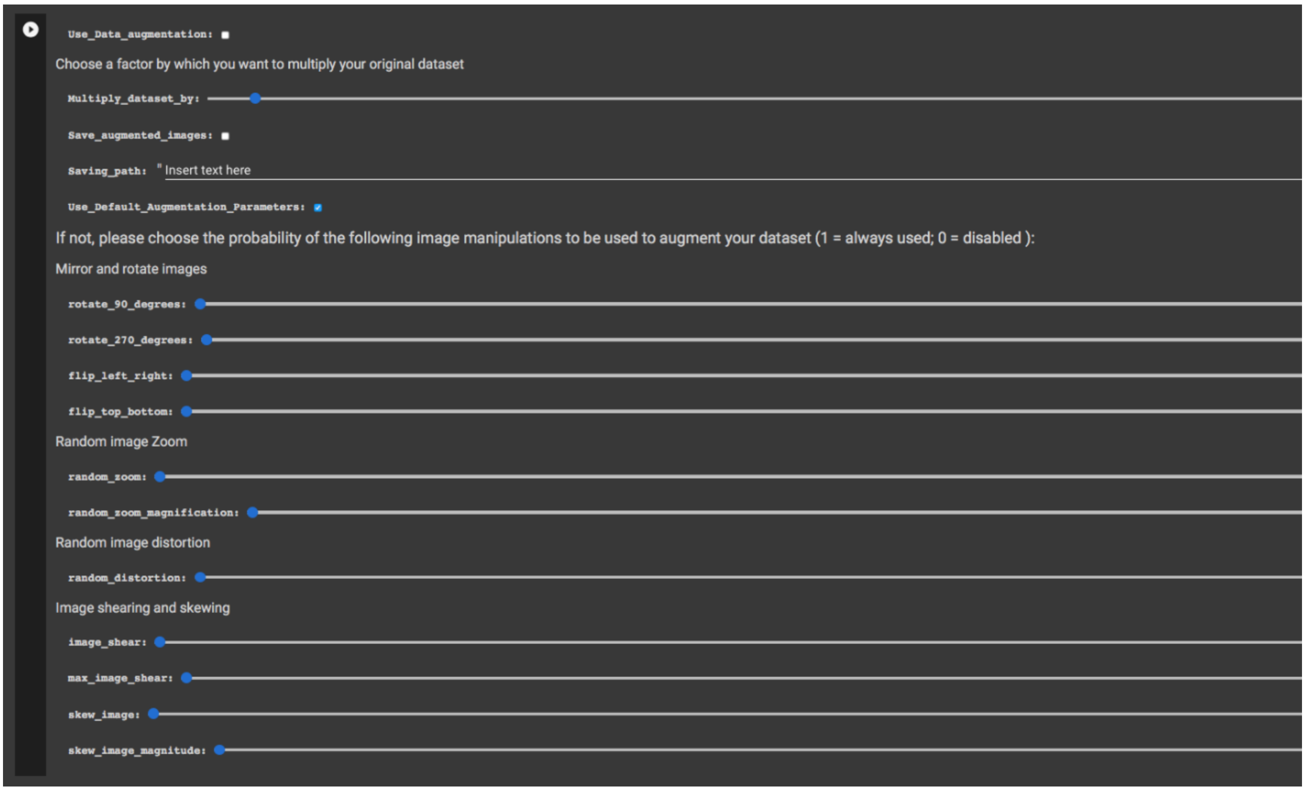

Figure 12: Setting the parameters for Data augmentation used in StarDist 2D. The multiplication factor applied to the training dataset can be selected as well as the probability of each transform to be used.

D.3. Transfer learning

Section 3.3 of the notebook gives access to transfer learning capabilities (see Supplementary Note 5 of our preprint for more details). Briefly, transfer learning allows the user to **load learned weights from a pre-trained model** into the new model prior to training. This allows the new training session to be initialised to an efficient starting point, **benefitting from features learned in the pre-trained model**. Consequently, this can have the advantage of **training more quickly** than with random weight initialisation (requiring fewer epochs) and may **lead to a more stable model**, depending on the pre-trained model used.

Since ZeroCostDL4Mic saves both the “best” (as defined by the set of weights that led to the minimum validation loss any time across the whole range of epochs) and the last set of weights (obtained after the last epoch of the training session), the user can choose to select either of these models to use as pre-trained weights for transfer learning. The corresponding learning rate is automatically loaded too, if possible.

Figure 13 shows an example of the transfer learning section that is available in the StarDist 2D notebook. A number of pre-trained models available online can be directly loaded.

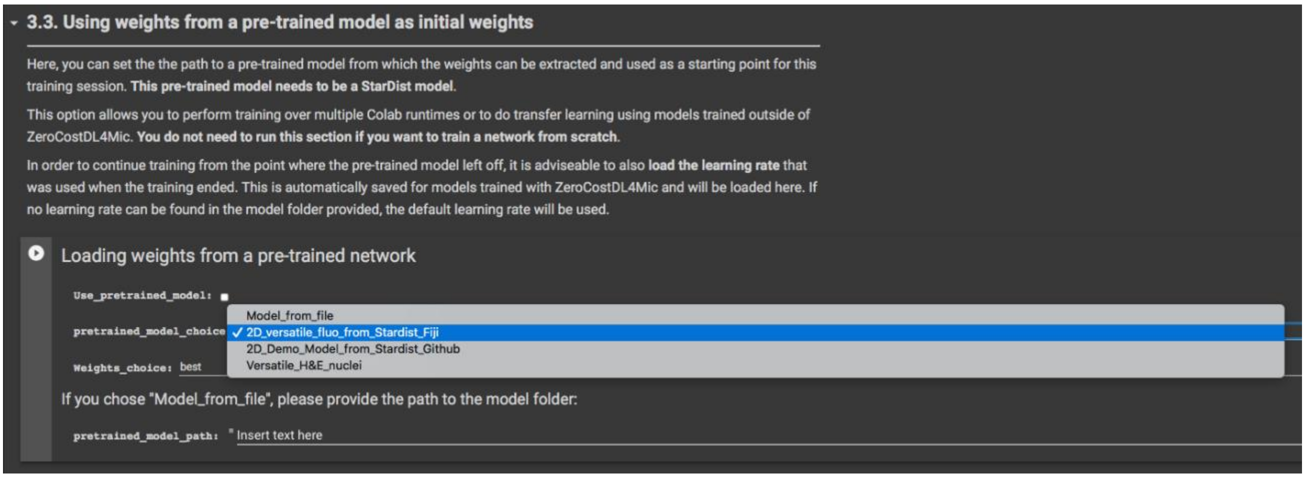

Figure 13: Transfer learning capabilities in StarDist 2D notebook. A pre-trained model can be used from a locally stored model (Model_from_file) or from StarDist 2D models made available online (e.g. 2D_versatile_fluo_from_Stardist_Fiji)

E. Training the model and performing quality control

E.1. Training

Here we go! Now, you can start the training by running the **4.2 Train the network** section (Figure 14). In some cases, the network may throw some minor warnings about TensorFlow versions, *this is not an issue and can be ignored*. We have enforced specific versions of TensorFlow in order to improve reliability of the notebooks.

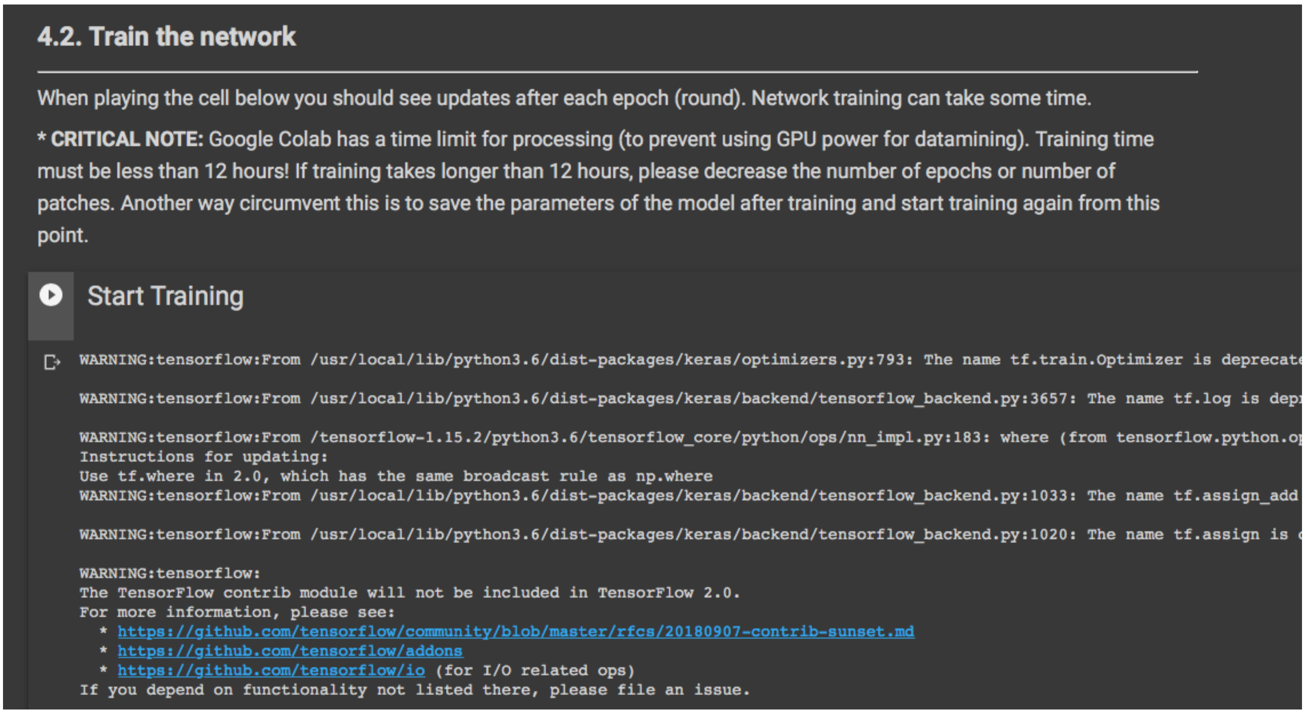

Figure 14: Training the model with the user-set parameters and training dataset.

This step can take a few minutes to a few hours depending on the network, training parameters and training dataset size. So be patient! Just ensure that the training time does not exceed 12 h, as this represents the current time limit of a Google Colab session. The time taken by each epoch is displayed for some networks as an output of the network training. This can be used to estimate how long the training will take (in Figure 15 shown as ~2s / epochs and 400 epochs, it should take about ~15min, so yes, you have time for a coffee). The total time taken for the training will be indicated once it is complete. **Pro tip: Keep in mind that you can run two Colab sessions in parallel**, so while one model is trained, you can set up another notebook and run it in parallel!

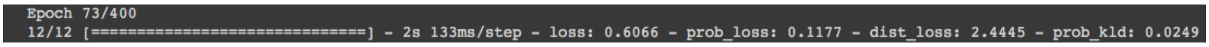

Figure 15: Network training output for each epoch, including the time taken for the epoch. This can help estimating the total time that the training will take.

After training is complete, **the trained model is automatically saved** into the **model_path** folder previously selected (Figure 16). This allows you to set the training running and ignore the risk of running into time-out issues after the training is over. The following sections of the notebooks can be run as a new Colab session if sections 1 and 2 of the notebook are run again.

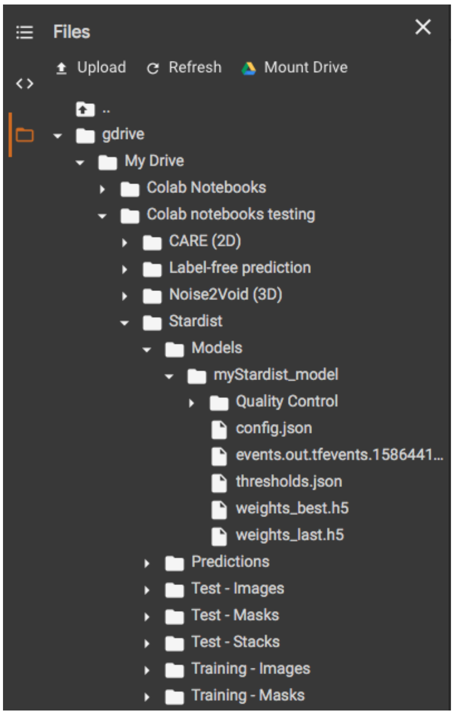

Figure 16: The newly trained model is automatically saved in the selected model folder.

You can download the trained model from your Google Drive for safekeeping or for sharing with another user (provided the limitations of using a pre-trained model highlighted in our bioRxiv paper[12]).

E.2. Quality control on the trained model

Section 5 of the notebook is very important! It allows you to perform some important quality controls on the trained model. This can be evaluated using two complementary approaches:

(1) Observe the evolution of the loss function as a function of training time. This allows for the inspection of the presence of over- or under-fitting of the model to the training dataset.

(2) Compute quantitative image metrics on the Quality control dataset (also often called "Test dataset" in the machine learning field), where the known ground truth can be compared to the prediction from this trusted dataset.

The quality control datasets should **not** be included in the training dataset during training, otherwise the network performance will be overestimated.

First, you will need to choose whether you are performing Quality control on the current trained model (as obtained in section 4), or whether you will be doing it on a model that was previously trained in a different session (Figure 17). For the latter, you will need to provide a model path and name.

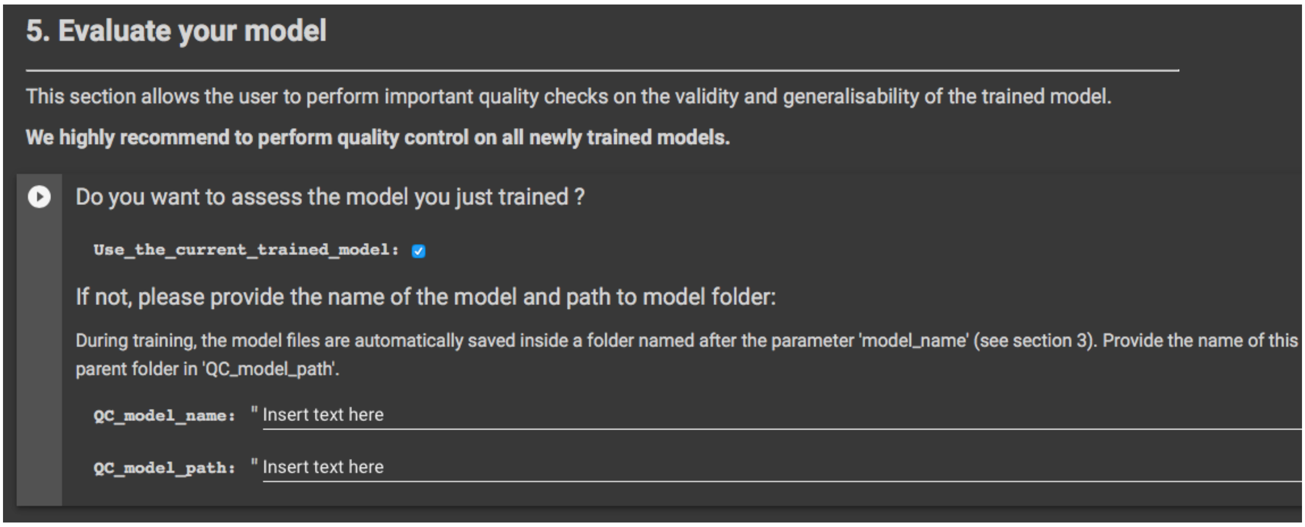

Figure 17: Choice of trained model to perform quality control. The path and name of a previously trained model can be given.

Figure 18 shows examples of loss function curves over training time, both from the training and validation dataset. If this doesn't make sense, please see our [Glossary page]() and also this review by Moen *et al.*[15], which explains how to interpret the curves very well.

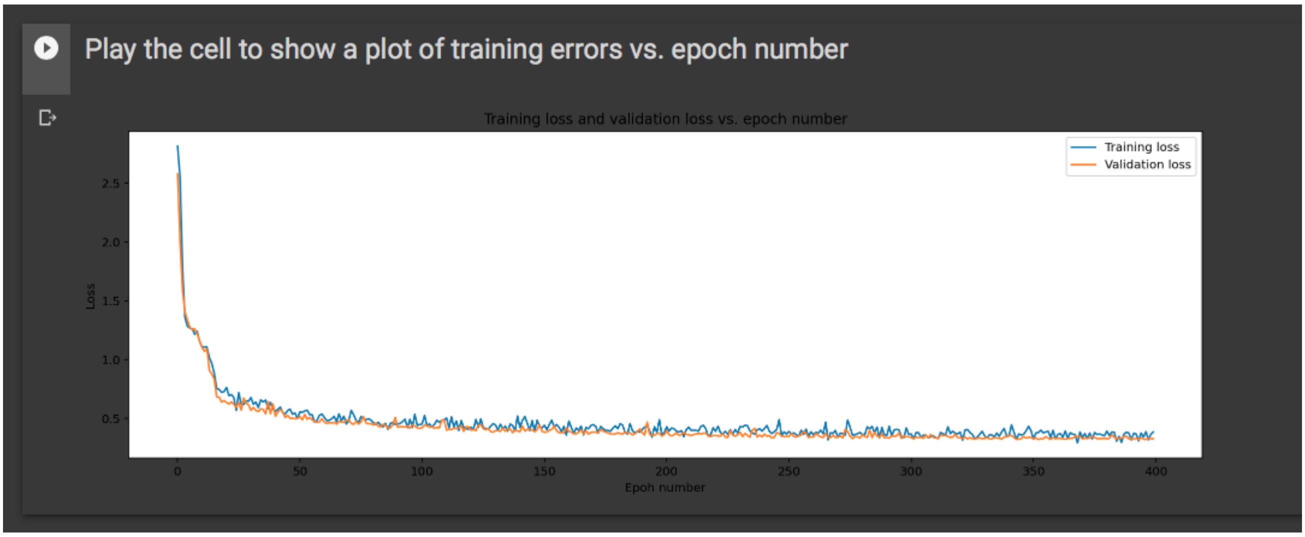

Figure 18: Evaluation of the model performance by inspection of the training and validation loss curves. A significant deviation over time between the two curves highlights a risk of overfitting of the model to the training dataset.

The second stage of quality control is to see whether the network is able to generalise to unseen dataset, using a quality control dataset. This is performed here by the user providing a dataset with the source images and the equivalent known ground truth targets. Depending on the type of network, we use **Root Squared Error** (RSE) and **Structural SIMilarity** (SSIM) or **Intersection over Union** (IoU) as metrics in order to visually and quantitatively assess whether the model can provide accurate output from unseen data.

Here, in the case of StarDist, because the model predicts a segmentation image, the metric used is Intersection over union (Figure 19). We describe the meaning of these metrics in detail in the Supplementary information of our paper.

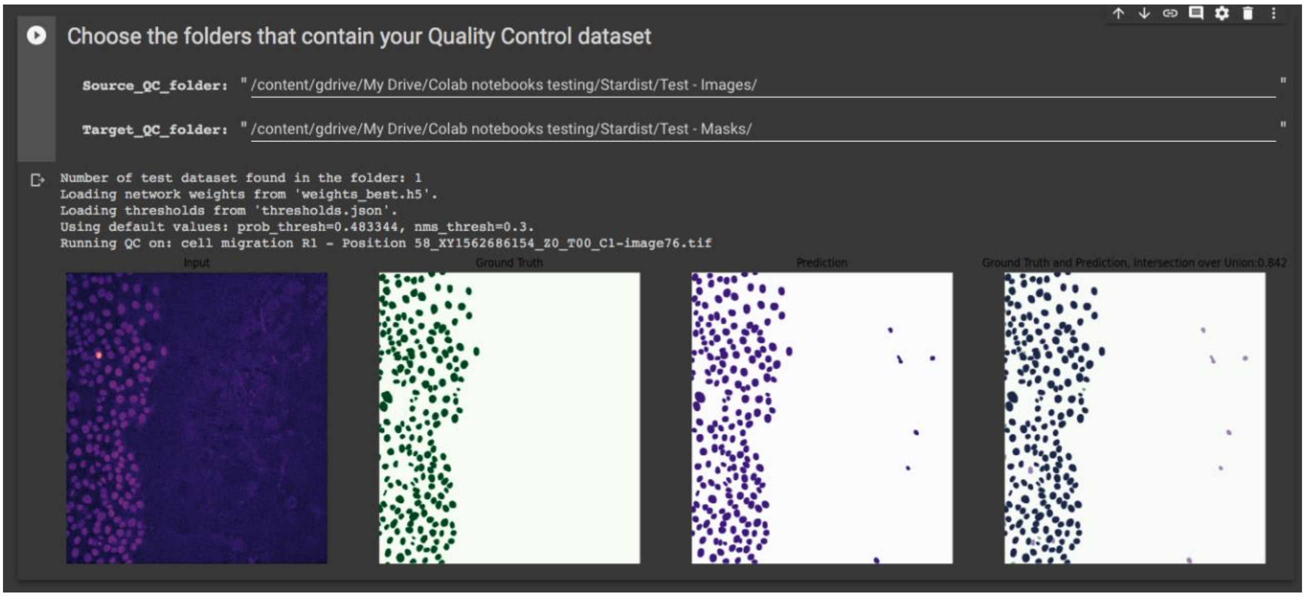

Figure 19: Quantitative assessment of a StarDist model performance using masks overlay and Intersection over Union metric. The image on the left is the source file to be segmented. The green mask represents the manually segmented ground-truth image, the magenta mask is the network prediction, the overlay shows the agreement and disagreement between the two. The Intersection over Union metric is indicated over the overlay image.

Once the metrics have been calculated the predictions and the metrics maps and indices are all automatically saved in the **model_path** folder in the *Quality Control* folder. You are strongly encouraged to take a look and analyse the data obtained on the test dataset in order to build confidence on the validity of the trained model to analyse the data in the Quality control dataset.

> **Important note**: When wondering if a pre-trained model may be suitable to analyse a new type of data, this section of Quality control can also be used to quantify and estimate if the model will perform well with this new type of data.

F. Using the trained model to obtain predictions on new data

The last step, available in **Section 6**, is what you have been waiting for the whole time: It is the generation of predictions from unseen data using the trained model. This can be performed by giving the path to the Google Drive directory containing the new unseen data that you wish to run the prediction on (Figure 20).

A different model from the currently loaded model can also be used by providing the name and path to the model to be used.

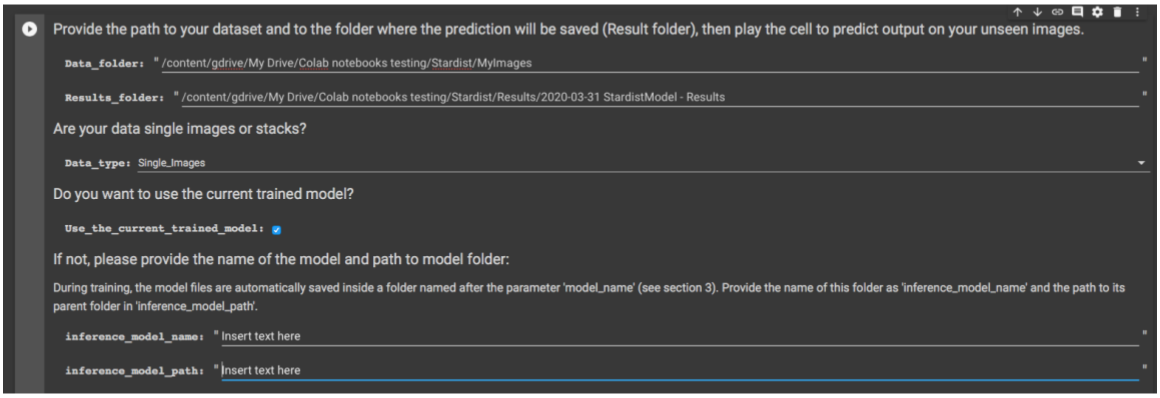

Figure 20: User input to run predictions on unseen data. In the particular case of StarDist, it is possible to run it directly on stacks (or on single images), and therefore there is an option to select this.

The notebook will show you an example prediction chosen at random from the set of data provided (Figure 21). In this example, we trained a StarDist model and the notebook shows an overlay of the original input image with the mask image obtained from the prediction.

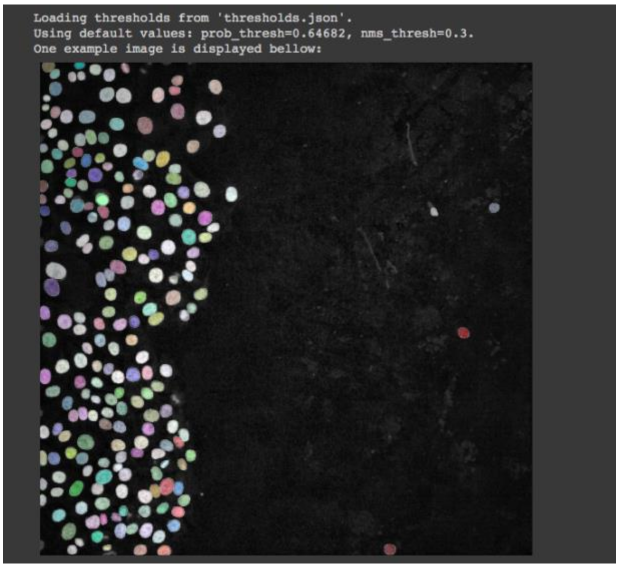

Figure 21: Prediction output display. The notebook chooses a random dataset to display for the visual assessment of the prediction.

The predictions obtained from the unseen data are now available in the Results folder as chosen earlier and can therefore be downloaded from your Google Drive for further analysis (Figure 22).

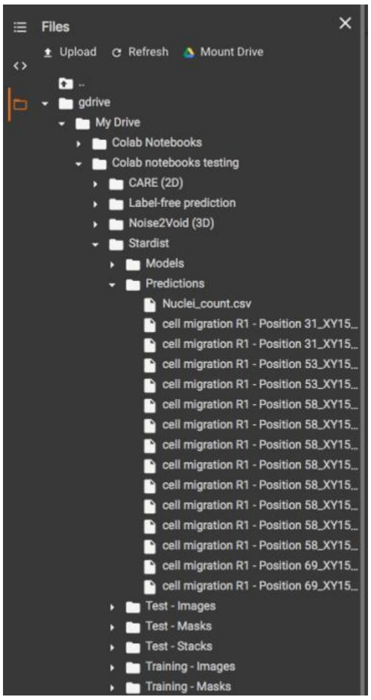

Figure 22: The predictions are automatically saved in the selected Results folder.

G. Final notes

Many thanks for trying out **ZeroCostDL4Mic**! Whether you find it useful, intuitive or difficult and buggy, we want to hear from you and always welcome constructive feedback. Feel free to report issues in the GitHub issue page or simply Tweet your results using #ZeroCostDL4Mic.

H. References

1. Ronneberger, O., Fischer, P. & Brox, T. U-Net: Convolutional Networks for Biomedical Image Segmentation. *ArXiv150504597 Cs* (2015).
2. Çiçek, Ö., Abdulkadir, A., Lienkamp, S. S., Brox, T. & Ronneberger, O. 3D U-Net: Learning Dense Volumetric Segmentation from Sparse Annotation. in *Medical Image Computing and Computer-Assisted Intervention – MICCAI 2016* (eds. Ourselin, S., Joskowicz, L., Sabuncu, M. R., Unal, G. & Wells, W.) vol. 9901 424–432 (Springer International Publishing, 2016).
3. Redmon, J. & Farhadi, A. YOLO9000: Better, Faster, Stronger. in 7263–7271 (2017).
4. Krull, A., Buchholz, T.-O. & Jug, F. Noise2Void - Learning Denoising from Single Noisy Images. *ArXiv181110980 Cs* (2019).
5. Schmidt, U., Weigert, M., Broaddus, C. & Myers, G. Cell Detection with Star-Convex Polygons. in *Medical Image Computing and Computer Assisted Intervention – MICCAI 2018* (eds. Frangi, A. F., Schnabel, J. A., Davatzikos, C., Alberola-López, C. & Fichtinger, G.) vol. 11071 265–273 (Springer International Publishing, 2018).
6. Weigert, M., Schmidt, U., Haase, R., Sugawara, K. & Myers, G. Star-convex Polyhedra for 3D Object Detection and Segmentation in Microscopy. 8 (2020).
7. Weigert, M. *et al.* Content-aware image restoration: pushing the limits of fluorescence microscopy. *Nat. Methods* **15**, 1090–1097 (2018).
8. Ounkomol, C., Seshamani, S., Maleckar, M. M., Collman, F. & Johnson, G. R. Label-free prediction of three-dimensional fluorescence images from transmitted-light microscopy. *Nat. Methods* **15**, 917–920 (2018).
9. Isola, P., Zhu, J.-Y., Zhou, T. & Efros, A. A. Image-to-Image Translation with Conditional

Adversarial Networks. *ArXiv161107004 Cs* (2018).

10. Zhu, J.-Y., Park, T., Isola, P. & Efros, A. A. Unpaired Image-to-Image Translation using Cycle-Consistent Adversarial Networks. *ArXiv170310593 Cs* (2018).
11. Nehme, E., Weiss, L. E., Michaeli, T. & Shechtman, Y. Deep-STORM: super-resolution single-molecule microscopy by deep learning. *Optica* **5**, 458 (2018).
12. von Chamier, L. *et al. ZeroCostDL4Mic: an open platform to simplify access and use of Deep-Learning in Microscopy*. http://biorxiv.org/lookup/doi/10.1101/2020.03.20.000133 (2020) doi:10.1101/2020.03.20.000133.
13. Arganda-Carreras, I. *et al.* Crowdsourcing the creation of image segmentation algorithms for connectomics. *Front. Neuroanat.* **9**, (2015).
14. Bloice, M. D., Roth, P. M. & Holzinger, A. Biomedical image augmentation using Augmentor. *Bioinformatics* **35**, 4522–4524 (2019).
15. Moen, E. *et al.* Deep learning for cellular image analysis. *Nat. Methods* **16**, 1233–1246 (2019).
