# Supplementary material for: Democratising deep learning for microscopy with ZeroCostDL4Mic
Source: Nat Commun. 2021 Apr 15;12:2276. doi: 10.1038/s41467-021-22518-0 (PMC8050272; doi:10.1038/s41467-021-22518-0)
Supplement: Supplementary file 15 — Description of Additional Supplementary Files [file 41467_2021_22518_MOESM15_ESM.pdf]

**Title: Supplementary Movie 1**:

**Description:** Full run-through of the workflow to obtain the notebooks and the provided test datasets as well as a common use of the notebook.

**Title: Supplementary Movie 2**:

**Description:** Representative results obtained using the ZeroCostDL4Mic notebooks and the provided test dataset.

**Title: Supplementary Movie 3:**

**Description:** Video highlighting how a 3D U-Net network trained using ZeroCostDL4Mic can be used to segment electron microscopy data. The raw data, as well as the training target (magenta) and the model predictions (green), are displayed. The visualisation of the 3D reconstruction was generated using Imaris.

**Title: Supplementary Movie 4**:

**Description:** Video highlighting how a StarDist model, trained using ZeroCostDL4Mic, can be used in conjunction with TrackMate[40] to track cell movement automatically. Raw data (Input), StarDist predictions as well as local cell tracks are displayed. Cells are DCIS.COM cells labelled with SiR-DNA and imaged using a spinning disk confocal microscope.

**Title: Supplementary Movie 5**:

**Description:** Video highlighting how a YOLOv2 model, trained using ZeroCostDL4Mic, can be used to detect and classify cell shapes in brightfield time-lapse data of MDA-MB-231 cells migrating on cell-derived matrices.

**Title: Supplementary Movie 6:**

**Description:** Example highlighting how a CARE network, trained using ZeroCostDL4Mic, can be used to denoise SIM live-cell imaging data. Here, a 3D CARE network was trained using SIM images of the actin cytoskeleton of DCIS.COM cells using fixed samples to denoise live-cell imaging data (see Online methods for details). Both input and the model predictions are displayed (representative single z plane).

**Title: Supplementary Movie 7:**

**Description:** Example highlighting how a Noise2Void 3D network, trained using ZeroCostDL4Mic, can be used to denoise live-cell imaging data. Movie of an ovarian carcinoma cell labelled with lifeact-RFP migrating on cell-derived matrices (labelled for fibronectin). Images were acquired using a spinning disk confocal microscope.

**Title: Supplementary Movie 8**:

**Description:** Example highlighting how a Noise2Void 3D network, trained using ZeroCostDL4Mic,

can be used to denoise microscopy data. Dataset #1: Live HeLa cells labelled with MitoTracker Red CMX Ros imaged with a VisiTech iSIM microscope, each frame represents a 20s interval. Dataset #2: Fixed HeLa cells immuno-labelled for microtubules and mitochondria imaged using a Leica SP8 confocal microscope.

**Title: Supplementary Movie 9**:

**Description:** Example highlighting how a Noise2Void 2D network, trained using ZeroCostDL4Mic, can be used to denoise live-cell imaging data. Video of a glioma cell endogenously labelled for paxillin-GFP, migrating on 9.6 kPa polyacrylamide hydrogel. Images were acquired using a spinning disk confocal microscope. Both the training source and model predictions are displayed.

**Title: Supplementary Movie 10**:

**Description:** Example highlighting how a Deep-STORM network, trained using ZeroCostDL4Mic, can be used to reconstruct high-density SMLM data. Reconstruction of a glial cell labelled with Phalloidin-AlexaFluor 647 (BIN4 dataset). The video shows the raw data, the wide-field equivalent data (obtained from binning the drift-corrected raw data), and the Deep-STORM reconstruction.

**Title: Supplementary Movie 11:**

**Description:** Example showcasing how ZeroCostDL4Mic notebooks can be used sequentially. Here we wanted to automatically track the migration pattern of DCIS.COM cells labelled with lifeact-RFP. Therefore, we first used pix2pix to “translate” the actin staining into nuclei staining and StarDist to detect the nuclei. From the StarDist prediction, cells were tracked automatically using TrackMate. In the video, the raw data (lifeact-RFP), the pix2pix predictions (Fake SiR-DNA), the StarDist predictions, and the TrackMate tracks are displayed.

**Title:** Supplementary Software

**Description:** The supplementary software provided contains the colaboratory notebooks used for this work as .ipynb files. This includes the following applications: content-aware image restoration (CARE) 2D, CARE 3D, StarDist 2D, StarDist 3D, U-Net 2D, U-Net 3D, Noise2Void 2D, Noise2Void 3D, Label-free prediction (fnet), pix2pix, cycleGAN, YOLOv2 and DeepSTORM.
